# Targeting the lipid metabolism proteins FASN and GPAM in alveolar type II cells decreases lung metastasis

**DOI:** 10.1158/2159-8290.CD-25-0191

**Published:** 2026-07-01

**Authors:** Xiao-Zheng Liu, Yulia Panina, Weigang Cai, Juan Fernández-García, Yiming Peng-Winkler, Jiadong Mao, Alina Dahlhaus, Hui-Chao Zhou, Ming Liu, Mélanie Planque, Johannes Ceuppens, Sebastian Igelmann, Xander Spotbeen, Jakub Idkowiak, Jonas Dehairs, Janine Theile, Konstantinos Axarlis, Kristien Borremans, Josephine Van Cauwenberge, Avinash Ghanate, Josep Tarragó-Celada, Alejandro Suárez-Bonnet, Richard Mitter, Vincen Wu, Paolo Inglese, James S. McKenzie, Rory T. Steven, Alex Dexter, Bin Yan, Jean-Luc Vorng, Zoltan Takats, Josephine Bunch, Ian S. Gilmore, Peter Carmeliet, Christine Desmedt, Ilaria Malanchi, Johannes V. Swinnen, Patricia Altea-Manzano, Kim-Anh Lê Cao, Johannes Meiser, Mariia Yuneva, Sarah-Maria Fendt

**Affiliations:** 1 Laboratory of Cellular Metabolism and Metabolic Regulation, VIB Center for Cancer Biology, VIB, Herestraat 49, 3000 Leuven, Belgium; 2 Laboratory of Cellular Metabolism and Metabolic Regulation, Department of Oncology, KU Leuven and Leuven Cancer Institute (LKI), Herestraat 49, 3000 Leuven, Belgium; 3 The Francis Crick Institute, London, NW1 1AT, UK; 4 Department of Gastroenterology, Hepatology and Infectious Diseases, University Hospital Dusseldorf, Dusseldorf, Germany; 5 Melbourne Integrative Genomics, School of Mathematics and Statistics, The University of Melbourne, Australia; 6 Cancer Metabolism Group, Department of Cancer Research, Luxembourg Institute of Health, 6A, Rue Nicolas-Ernest Barblé, 1210 Luxembourg, Luxembourg; 7 Laboratory of Angiogenesis and Vascular Metabolism, Department of Oncology, KU Leuven, Leuven, B-3000, Belgium; 8 Laboratory of Angiogenesis and Vascular Metabolism, Center for Cancer Biology, VIB, Leuven, B-3000, Belgium; 9 Spatial Metabolomics Expertise Center, VIB Center for Cancer Biology, VIB, Herestraat 49, 3000 Leuven, Belgium; 10 Laboratory of Lipid Metabolism and Cancer, Leuven Institute for Single Cell Omics (LISCO) and Leuven Cancer Institute (LKI), Department of Oncology, KU Leuven, Leuven, Belgium; 11 Tumour–Host Interaction Laboratory, The Francis Crick Institute, London, UK; 12 Laboratory for Translational Breast Cancer Research, Department of Oncology, KU Leuven, Herestraat 49, 3000 Leuven, Belgium; 13 Gynecological Oncology Unit, Department of Oncology, UZ Leuven, Leuven, Belgium; 14 Experimental Histopathology Platform. The Francis Crick Institute, London, NW1 1AT, UK; 15 Pathobiology and Population Sciences, Royal Veterinary College, Hatfield, AL9 7TA, UK; 16 Faculty of Medicine, Department of Metabolism, Digestion and Reproduction, Imperial College London, South Kensington Campus, London, SW7 2AZ, UK; 17 National Physical Laboratory, Hampton Road, Teddington, United Kingdom, TW11 0LW; 18 Center for Biotechnology, Khalifa University of Science and Technology, Abu Dhabi, United Arab Emirates; 19 Laboratory of Metabolic Regulation and Signaling in Cancer, Andalusian Molecular Biology and Regenerative Medicine Centre (CABIMER) - CSIC- University of Seville - University Pablo de Olavide, Seville, Spain

## Abstract

Cancer cells that seed in the lung require lipids often produced by alveolar type II (AT2) cells. However, whether overt metastases depend on AT2 cell-derived lipids and whether AT2 cells can be targeted to reduce metastasis growth remains unknown. We discovered that breast cancer-derived lung metastases stimulate the proliferation of AT2 cells in their vicinity and reprogram them into lipid feeder cells in mice and patients using spatial analysis. Mechanistically, the metastasis secretome activates the transcription factor sterol regulatory element-binding transcription factor 1 (SREBP-1) in AT2 cells, enhancing the expression of key *de novo* lipid synthesis genes including fatty acid synthase (*FASN*) and glycerol-3-phosphate acyltransferase 1 (*GPAM*). Deleting *Fasn* selectively in AT2 cells or targeting FASN and GPAM systemically significantly impairs lung metastasis growth in mice. In summary, we discovered that overt metastases reprogram AT2 cells and that targeting the lipid metabolism of AT2 cells impairs metastasis growth.

## Introduction

Metastasis formation is the leading cause of cancer related deaths (1), with the lung being a common metastatic site in many cancers, including breast cancer (2). Despite significant advancements in targeted cancer therapies, the clinical management of metastatic breast cancer remains challenging (3). Once metastases are established, therapeutic options become limited, and the disease is generally considered incurable (4). This lack of curative treatments for metastatic cancer underscores the urgent need for new therapeutic strategies.

A critical aspect of metastasis seeding is the formation of the pre-metastatic niche, which is a specialized microenvironment within distant organs that supports the survival and proliferation of disseminated cancer cells (5). Resident cells within this niche, such as fibroblasts, immune cells, and endothelial cells, play a pivotal role in promoting metastasis seeding (6–8). These cells interact with disseminated cancer cells, providing essential signals and factors that facilitate their survival, dormancy, or reactivation (9,10). In this respect, it has been found that cancer-associated fibroblasts can remodel the extracellular matrix to create a metastasis-supportive environment (6,7), while immune cells like alveolar macrophages can induce dormancy in disseminated breast cancer cells (10). Moreover, lung-resident alveolar type II (AT2) cells, which are responsible for synthesizing and secreting the lipid-rich surfactant of the lung (11), have been shown to enable metastatic seeding by providing lipids to the arriving cancer cells (12). Additionally, it has been reported that AT2 cells promote stem-like features in cancer cells and enhance their initiation capacity in the metastatic niche (13). However, it remains largely elusive whether AT2 cells play a role once metastases have established and grow in the lung.

Here, we studied the interaction between AT2 cells and breast cancer-derived lung metastases by profiling the metabolome and transcriptome of metastatic lungs with temporal and spatial resolution. We discovered that, in the vicinity of metastases, AT2 cells are reprogramed into lipid feeder cells, and that targeting the enzymes FASN and GPAM in AT2 cells impairs metastasis growth.

## Results

### AT2 cells and surfactant lipids are enriched in the vicinity of lung metastases from patients with breast cancer

**Figure 1:**
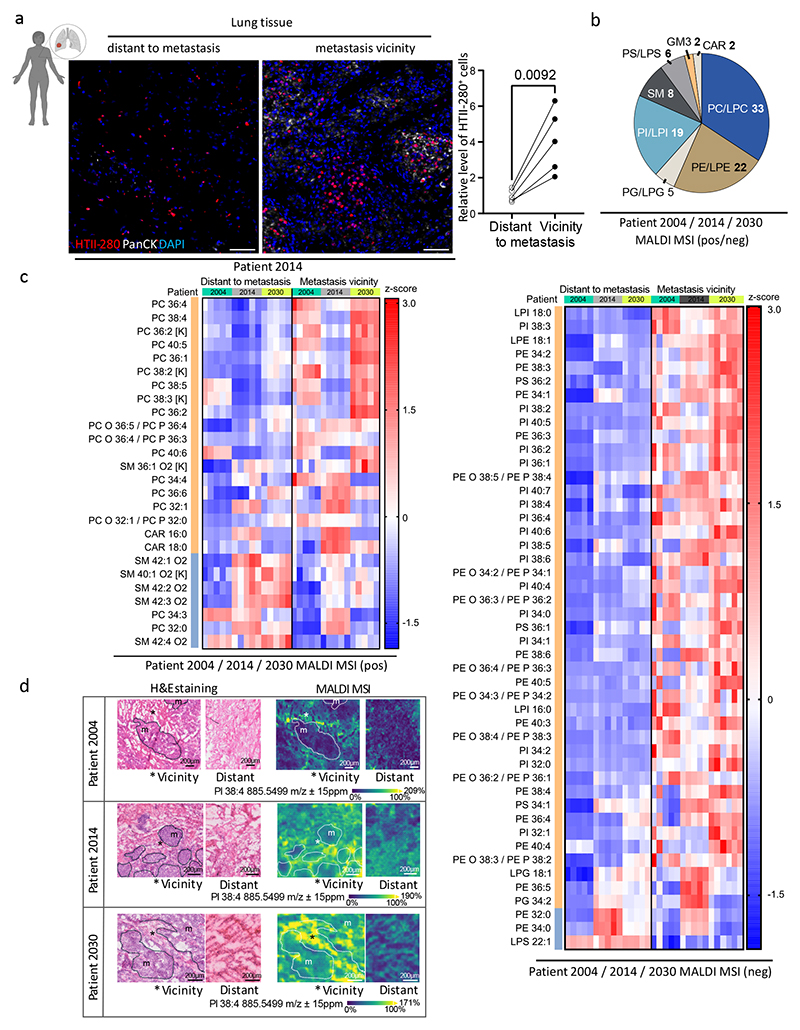
AT2 cells and surfactant lipids are enriched in the vicinity of lung metastases from patients with breast cancer a. Representative Opal Multiplex immunohistochemistry (IHC) staining for HTII-280 (red), PanCK (white), and DAPI (blue) in the lung tissue distant to metastasis and lung tissue in the vicinity of metastasis from breast cancer patient 2014 of the UPTIDER program. Scale bars represent 50 μm. n = 5 breast cancer patients (3 areas per patient per condition, for patient 2009 two regions were selected due to the limited availability of distant tissue in the section) were examined. Quantification of all patients is shown on the right panel. Graphs show relative level of AT2 cells. Statistical significance was determined using paired t-tests. b. Pie charts showing the number of detected lipids in metastatic lungs from breast cancer patient 2004,2014 and 2030 of the UPTIDER program. Pos and neg represent the positive and negative ion mode in the MALDI-MSI detection settings, respectively. Abbreviations: PC, phosphatidylcholines; LPC, lysophosphatidylcholine; PE, phosphatidylethanolamines; LPE, lysophosphatidylethanolamine; PG, phosphatidylglycerol; LPG, lysophosphatidylglycerol; PI, phosphatidylinositols; LPI lysophosphatidylinositol; SM, sphingomyelin; GM3, monosialodihexosylganglioside; CAR, acylcarnitines. c. Heatmaps showing z-score distributions of region-aggregated, scaled total ion count (TIC)-normalized abundances of lipids found to be significantly altered between lung tissues distant to metastases and metastasis-vicinity regions, as measured by positive-polarity (left) and negative-polarity (right) MALDI-MSI in metastatic lung tissue samples from 3 independent patients. Each row represents a distinct lipid species, and each column represents each of the individual metastasis-distant or metastasis-vicinity regions annotated for each of the different patients. The color code indicates z-scores based on region-aggregated, scaled total ion count (TIC)-normalized abundances for each lipid species in each of these regions (see Methods), with the z-score transformation performed on a per-lipid basis, across all shown patient/region combinations. Lipid-level alteration magnitudes and their statistical significance for each polarity were determined based on a linear model applied on a per-lipid basis, with TIC-normalized abundances for each lipid species first scaled relative to their maximum value within each tissue section, then aggregated within each individual region (n = 5-6 regions per patient for each of lung tissue distant to metastasis and metastasis vicinity), and finally modeled as a function of the region type (treated as a categorical predictor), with the patient of origin treated as a blocking variable (see Methods). Only those lipid species showing FDR-adjusted p-values below 0.05 for the *metastasis vicinity vs lung tissue distant to metastasis* contrast are shown in the plots. Orange/blue bars indicate metabolites that are significantly higher/lower in the metastasis vicinity compared to lung tissue distant to the metastasis, respectively. d. Representative total ion count-normalized MALDI-MSI ion images of metastatic lung tissues from breast cancer patient 2004, 2014 and 2030 of the UPTIDER program, alongside an optical image of the H&E staining of the same tissue section. Lung metastases (m) are outlined with dash lines on both H&E and MSI images, and * indicate the vicinity of metastases. Scale bars: 200 μm. The same representative images, along with overview images, are also shown in Supplementary Figure 1b. (a. Created with BioRender.com)

To investigate the role of AT2 cells in an established metastatic disease, we analyzed metastatic lung tissues from five patients with breast cancer enrolled in the postmortem tissue donation program UPTIDER (NCT04531696, KU/UZ Leuven). We used HTII-280 as AT2 cell marker (14) and PanCK as a cancer cell marker. We determined the AT2 cell number and distribution in the vicinity of metastases (100-200μm from the metastasis interface) compared to non-cancerous distal-lung areas (no visible metastases present within 250μm of the selected regions) (Supplementary Figure 1a). We discovered that AT2 cells were significantly enriched in the vicinity of metastases from patients with breast cancer relative to distal non-cancerous regions of the lung (Figure 1a).

Since AT2 cells are the main lipid producers of the lung, we asked whether the enrichment of AT2 cells in the vicinity of metastases increased the lipid availability in these tissue regions. To gain a spatial resolution of metabolism, we used matrix-assisted laser desorption/ionization (MALDI) mass spectrometry imaging (MSI) and profiled lipids including surfactant lipids in the metastatic lungs of three patients (assigning vicinity and distal regions relative to 3-5 metastases within each tissue sample). Across the three patients we commonly detected 97 lipid species (Figure 1b), with phosphatidylcholine (PC), phosphatidylethanolamine (PE) and phosphatidylinositol (PI) lipids, which are frequently found in lung surfactant, showing a significant increase in the vicinity of metastases compared to distal non-cancerous regions and a similar spatial distribution to that of AT2 cells (Figure 1c-d, Supplementary Figure 1b). Based on these data we concluded that AT2 cells and lipids including surfactant lipids are enriched in the vicinity of lung metastases from patients with breast cancer.

### AT2 cells and surfactant lipids co-localize in the vicinity of metastases from mice

**Figure 2:**
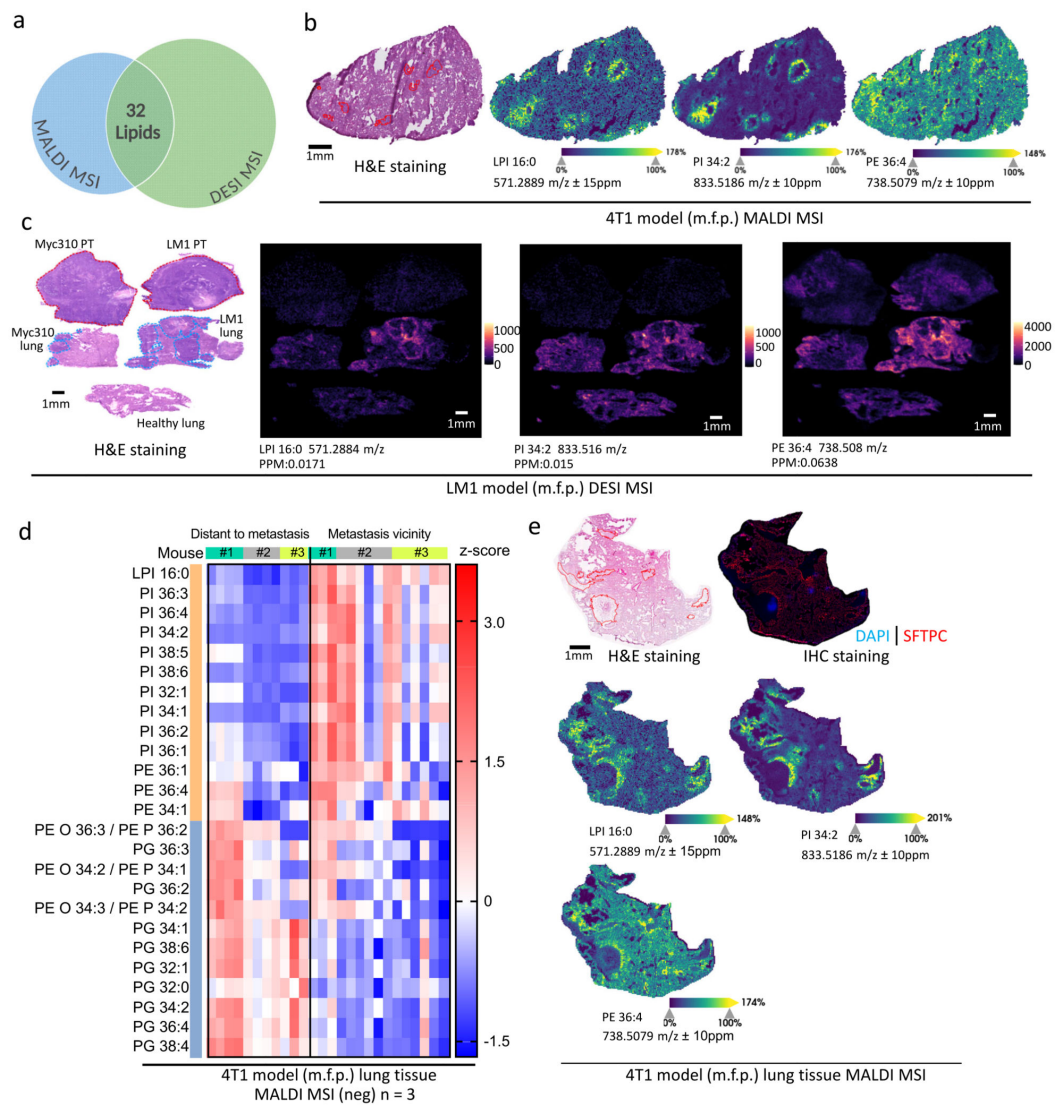
AT2 cells and surfactant lipids co-localize in the vicinity of metastases from mice a. Venn diagram depicting the number of overlapping lipid ions detected by both MALDI-MSI on 4T1 (m.f.p.) derived lung metastases and DESI-MSI LM1 (m.f.p.) derived lung metastases. b. Representative total ion count-normalized MALDI-MSI ion images of metastatic lung tissues from 4T1 m.f.p. injection derived lung metastasis, alongside an optical image of the H&E staining of the same tissue section. Lung metastases are outlined with red lines on the H&E staining. Scale bars represent 1mm. c. Representative DESI-MSI ion images of primary mammary gland tumors, corresponding metastatic and healthy lungs, alongside an optical image of the H&E staining of the same tissue section. Lung metastases are outlined with blue dash lines on the H&E staining. Scale bars represent 1mm. d. Heatmap showing z-score distributions of region-aggregated, scaled total ion count (TIC)-normalized abundances for lipid species among the 32 identified in the overlap area of Figure 2a and further found to be significantly altered between lung tissues distant to metastases and metastasis-vicinity regions, as measured by negative-polarity MALDI-MSI in metastatic lung tissue samples from 3 mice with 4T1 (m.f.p.) derived lung metastases. Each row represents a distinct lipid species, whereas each column represents each of the individual metastasis-distant or metastasis-vicinity regions annotated for each of the different mice. The color code indicates z-scores based on region-aggregated, scaled total ion count (TIC)-normalized abundances for each lipid species in each of these regions (see Methods), with the z-score transformation performed on a per-lipid basis, across all shown mouse/region combinations. Lipid-level alteration magnitudes and their statistical significance were determined based on a linear model applied on a per-lipid basis, with TIC-normalized abundances for each lipid species first scaled relative to their maximum value within each tissue section, then aggregated within each individual region (n = 3-6 regions per mouse for each of lung tissue distant to metastasis and metastasis vicinity), and finally modeled as a function of the region type (treated as a categorical predictor), with the mouse of origin treated as a blocking variable (see Methods). Only those lipid species showing FDR-adjusted p-values below 0.05 for the *metastasis vicinity vs lung tissue distant to metastasis* contrast are shown in the plots. Orange/blue bars indicate metabolites that are significantly higher/lower in the metastasis vicinity compared to lung tissue distant to the metastasis, respectively. e. Representative total ion count-normalized MALDI-MSI ion images of metastatic lung tissues from 4T1 m.f.p. injection derived lung metastasis, alongside an optical image of the H&E staining and an IHC staining for SFTPC (red) and DAPI (blue) of the same tissue section. Lung metastases are outlined with red lines on the H&E staining. Scale bars represent 1mm.

We next used MSI to assess the spatial distribution of lipids in metastatic lungs of mice in relation to the enrichment of AT2 cells in the vicinity of the metastases. We detected 86 common lipid species across three metastatic mouse lungs (4T1 cells, mammary fat pad (m.f.p.) 22 days) using MALDI-MSI, and 119 common lipid species across two metastatic mouse lungs (LM1 cells derived from the MMTV-MYC mouse model, m.f.p. 21-28 days, Supplementary Figure 2a) using desorption electrospray ionization (DESI)-MSI (Supplementary Figure 2b-c). Across the two models and analytical methods, 32 surfactant lipid species (Figure 2a), including PI, lysophosphatidylinositol (LPI), PE and phosphatidylglycerol (PG), were commonly identified (Figure 2b-c). Next, we spatially analyzed the commonly detected lipids (assigning vicinity and distal regions relative to 3-5 metastases within each tissue sample). Like in patient samples, many of the PI and PE lipids showed a significantly increased abundance in the vicinity of metastases compared to non-cancerous lung tissue distal to the metastases in both the m.f.p. and intravenous (i.v.) injection models (Figure 2d, Supplementary Figure 2d-e). This observation was further confirmed by an accumulation of lipids specifically in the vicinity of metastatic lesions (assigned based on the high nucleotide fragment signal m/z 134.0471) (15) using the high-resolution MSI platform orbitrap secondary ion mass spectrometry (OrbiSIMS) (Supplementary Figure 2f). To further assess whether the lipid-enriched areas co-localized with AT2 cell-enriched regions, we combined MALDI-MSI with immunohistochemistry staining for the AT2 cell marker surfactant protein C (SFTPC) (16) on the same tissue section. We observed that AT2 cells were particularly enriched in areas with increased lipid levels in the vicinity of 4T1-derived lung metastases (Figure 2e). Thus, our findings demonstrate that AT2 cells display a strong spatial association with increased lipid levels in the vicinity of metastases from mice.

### Disease progression increases the enrichment of AT2 cells in the vicinity of metastases

**Figure 3:**
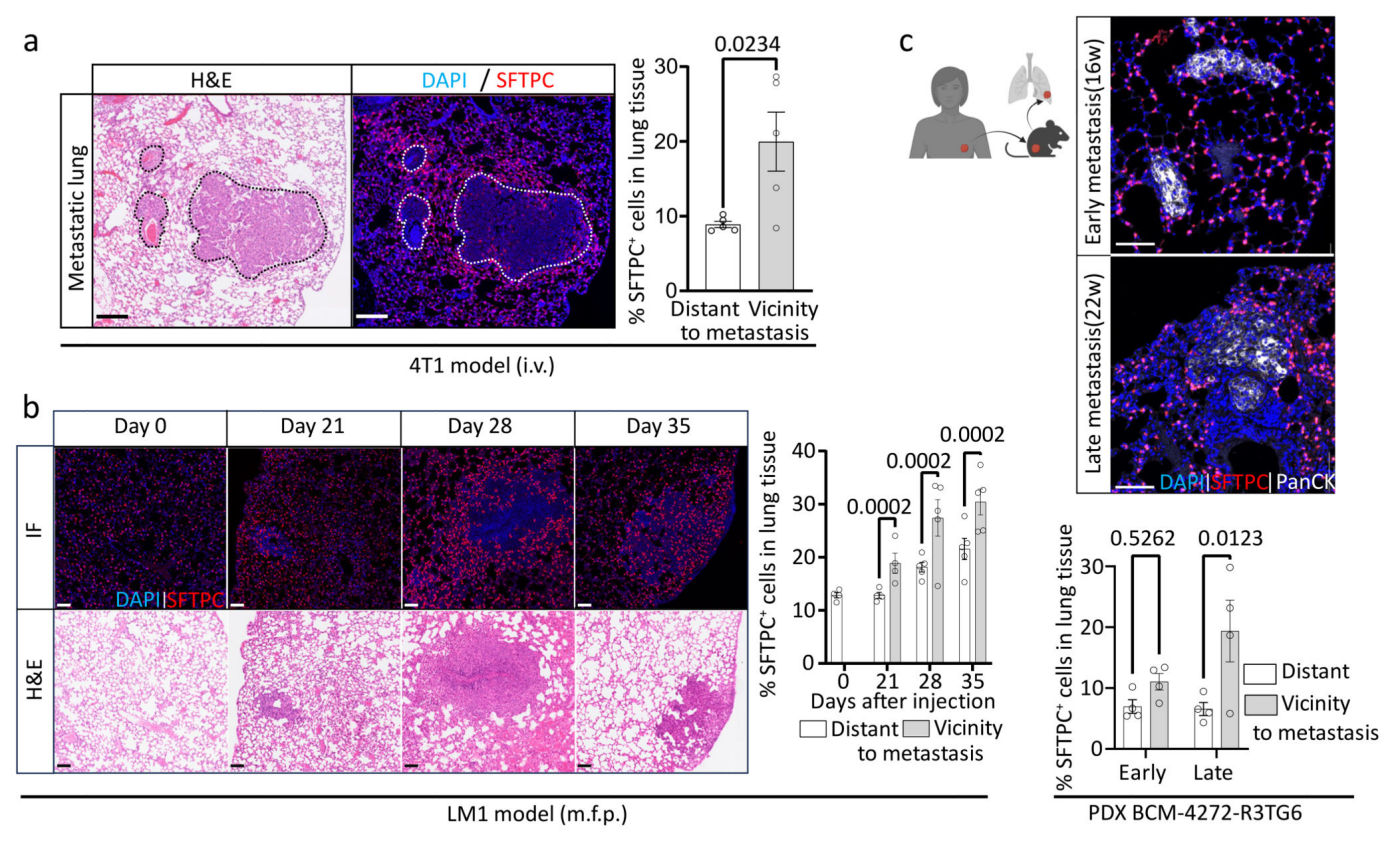
Metastases progression increases the enrichment of AT2 cells a. Representative IHC staining for SFTPC (red) and DAPI (blue) in lung metastases derived from 4T1 intravenous injection (i.v.), alongside an optical image of the H&E staining of the same tissue section. Lung metastases are outlined with dash lines. Scale bars represent 200 μm. The percentage of SFTPC^+^ cells is shown as mean ± SEM of n=5 mice on the right panel. Statistical significance was determined using unpaired two-tailed t-test. b. Representative IHC staining for SFTPC (red) and DAPI (blue) in longitudinally collected metastatic lung tissues (at day 0, 21,28 and 35 post-injection) derived from LM1 m.f.p. injected mouse models, alongside optical images of the H&E staining of the consecutive sections. Scale bars represent 100 μm. The percentage of SFTPC^+^ cells, quantified either in the vicinity of metastasis (200μm from a metastatic lesion border) or in a distant area to metastasis (>500μm from a metastatic lesion border) is shown as mean ± SEM of biological replicates (n = 4 of mice for day 0 and 21, n = 5 of mice for day 28 and 35) on the right panel. Statistical significance was determined using two-way ANOVA with Šídák multiple comparison test. c. Representative IHC staining of SFTPC (red), PanCK (white), and DAPI (blue) in early (16 weeks) and late (22 weeks) metastatic lungs from orthotopic (m.f.p.) TNBC PDX models. Scale bars represent 100 μm. The percentage of SFTPC^+^ cells is shown as mean ± SEM of n=4 PDX mice on the right panel. Statistical significance was determined using two-way ANOVA with Šídák multiple comparison test. (c. Created with BioRender.com)

Next, we investigated whether the growth of lung metastases can directly impact AT2 cells, or whether, as previously shown (12), the formation of a pre-metastatic niche by the primary tumor is necessary. For this, we generated lung metastases without a primary tumor, by i.v. injecting 4T1 and EMT6.5 cancer cells into mice. Subsequently, we assessed the distribution of AT2 cells in the metastatic lungs. We observed an enrichment of AT2 cells in the vicinity of metastases compared to distal non-cancerous regions of the lungs (Figure 3a, Supplementary Figure 3a) by assessing SFTPC, mucin 1 (MUC1) (17) and lysosomal associated membrane protein 3 (LAMP3, also known as DC-LAMP) (18) triple-positive AT2 cells (Supplementary Figure 3b). Thus, we concluded that the presence of metastases is sufficient to enrich AT2 cells in their vicinity.

We subsequently asked whether the AT2 cell enrichment in the vicinity of lung metastases occurs progressively during metastasis growth. To answer this, we injected LM1 cells into the m.f.p. of FVB/NJ mice. Subsequently, we collected lung samples longitudinally to monitor the changes in AT2-cell number and localization in response to the growth of the metastases (day 0, 21, 28 and 35 post-m.f.p. injection). Strikingly, we observed a gradual increase in the number of AT2 cells in the vicinity of metastases as the latter progressed (Figure 3b). This enrichment of AT2 cells in the vicinity of metastases was confirmed in an independent 4T1 breast cancer mouse model with late-stage lung metastases (22 days post-m.f.p. injection; Supplementary Figure 3c). Therefore, we concluded that AT2 cells progressively enrich in the vicinity of lung metastases from syngeneic mouse models.

To relate this spatiotemporal information from mouse breast cancer metastases to patients, we used tissue samples from a patient-derived xenograft (PDX) mouse model collected at an early-stage (16 weeks) compared to a late-stage (22 weeks) lung metastatic disease (19,20). Like mouse breast cancer-derived metastases, we found a homogenous distribution of AT2 cells at the early-stage metastatic disease (week 16), which progressed to a significant enrichment of AT2 cells in the vicinity of metastases at the late-stage metastatic disease (22 weeks, Figure 3c) in the PDX mouse model. This provides evidence that the temporal enrichment of AT2 cells in the vicinity of breast cancer-derived lung metastases is reflective of the metastatic disease in patients.

### Proliferation drives the AT2 cell enrichment in the vicinity of metastases

**Figure 4:**
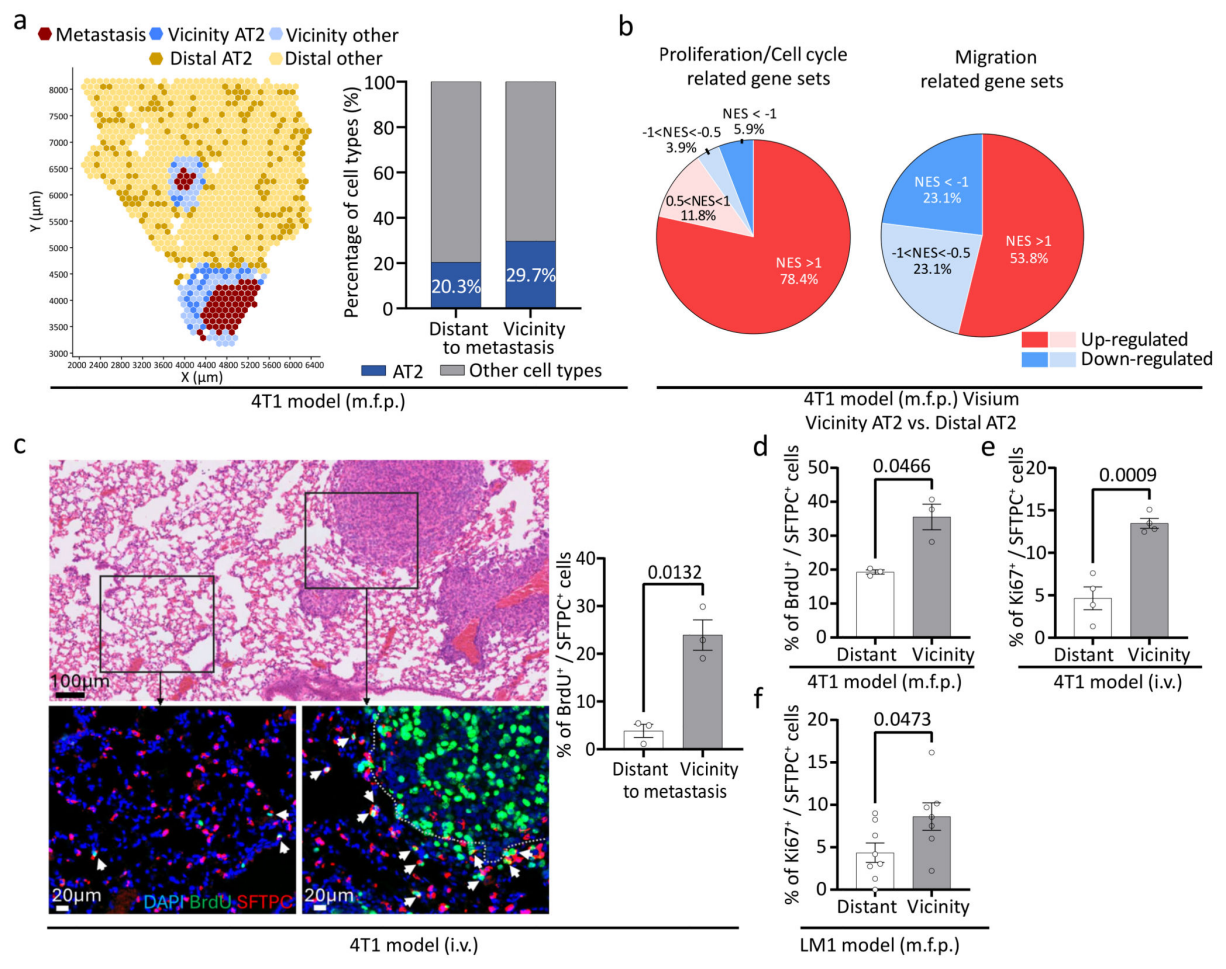
Proliferation drives the AT2 cell enrichment in the vicinity of metastases a. Visium spatial plot of metastatic lung tissue from the 4T1 m.f.p. injection model (left). Unsupervised clustering based on PhiSpace scores identified 5 spatial niches, which were further annotated to 3 region types: metastasis-core, metastasis-vicinity, and metastasis-distal regions. AT2 cell–enriched spots were determined as those Visium spots presenting PhiSpace AT2-cell scores among the top 20% percentile of all spots. The latter are shown in darker color shades for both the metastasis-vicinity and metastasis-distal regions (no AT2 cell-enriched spots were found within the metastasis-core region). The proportion of AT2-enriched spots in each region type is indicated by the bar plot (right). b. Pie charts showing the proportions of strongly/mildly up- and down-regulated gene sets related to proliferation/cell cycle (left) and migration (right), based on GSEA-derived normalized enrichment scores (NES) determined from the Visium data in (Figure 4a), comparing AT2 cell-enriched spots in metastasis-vicinity *vs* metastasis-distal regions. c. Representative H&E staining and immunofluorescence (IF) staining of SFTPC (red), BrdU (green), and DAPI (blue) in metastatic lungs from 4T1 (i.v.) model. Mice were injected intraperitoneally (i.p.) with two doses of BrdU in saline (50 mg/kg) 30 hours and 6 hours prior to euthanasia. Lung metastases are outlined with white dash line, and BrdU^+^AT2 cells are indicated by arrows. Scale bars: 100 μm (H&E) and 20 μm (IF). The percentage of BrdU^+^ AT2 cells is shown as mean ± SEM of n=3 mice (right). Statistical significance was determined using unpaired two-tailed t-test. d. Quantification of IF staining of SFTPC and BrdU in metastatic lungs from 4T1 (m.f.p.) model. Mice were injected i.p. with two doses of BrdU (50 mg/kg) before euthanasia. The percentage of BrdU^+^ AT2 cells is shown as mean ± SEM of n=3 of mice. Statistical significance was determined using unpaired two-tailed t-test. e. Quantification of IHC staining of SFTPC and proliferation marker Ki67 in metastatic lungs from 4T1 (i.v.) models. The percentage of Ki67^+^ AT2 cells is shown as mean ± SEM of n=4 mice for the 4T1 (i.v.) model. Statistical significance was determined using unpaired two-tailed t-test. f. Quantification of IF staining of SFTPC and proliferation marker Ki67 in metastatic lungs from LM1 (m.f.p.) models. The percentage of Ki67^+^ AT2 cells is shown as mean ± SEM of n=8 mice (one sample section did not contain visible metastasis; therefore, a random metastasis-free area was selected and included in the distant group) for the LM1 (m.f.p.) model. Statistical significance was determined using unpaired two-tailed t-test.

Next, we asked whether the enrichment of AT2 cells in the vicinity of metastases is driven by increased AT2-cell proliferation or, alternatively, by migration of the latter towards the metastases. To answer this, we performed Visium spatial transcriptomics on a metastatic lung from the 4T1 m.f.p. injection model, and analyzed the data with the PhiSpace pipeline (21), which allowed us to identify distinct spatial niches, including metastasis-core, metastasis-vicinity and metastasis-distal regions (Figure 4a, Supplementary Figure 3d). Subsequently, we ranked all Visium spots according to their PhiSpace-derived AT2 scores, calculated based on AT2 markers derived from the matching scRNA-seq reference dataset (22), which allowed us to infer the locations of AT2-enriched spots. In line with the immunostaining for AT2 cell markers, we observed an increased proportion of AT2-enriched spots in the vicinity of the metastases relative to metastasis-distal regions (Figure 4a). Subsequently, we assessed the enrichment of proliferation and migration gene signatures in AT2-enriched spots in the vicinity of the metastases relative to those in metastasis-distal regions. We found that the AT2-enriched spots in the vicinity of the metastases showed an enrichment of gene expression signatures indicative of enhanced proliferation, while gene signatures suggesting increased migration were only mildly enriched in those same spots (Figure 4b). To confirm the spatial transcriptomics predictions, we assessed AT2 cell proliferation by *in vivo* 5-bromo-2’-deoxyuridine (BrdU) incorporation (23) in both the 4T1 m.f.p. and i.v. models (two 50 mg/kg doses of BrdU intraperitoneally (i.p.) injected prior to euthanasia). In line with the spatial transcriptomics analysis, BrdU incorporation was significantly higher in AT2 cells located in the vicinity compared to the distance of the metastases (Figure 4c-d), which was further confirmed by co-staining metastatic lungs for AT2 cells and the proliferation marker Ki67 in the 4T1 i.v. and LM1 m.f.p. models (Figure 4e-f). Thus, we concluded that AT2 cells in the vicinity of mouse lung metastases display increased proliferation.

### The metastasis secretome reprograms AT2 cell lipid metabolism by activating SREBP-1

**Figure 5:**
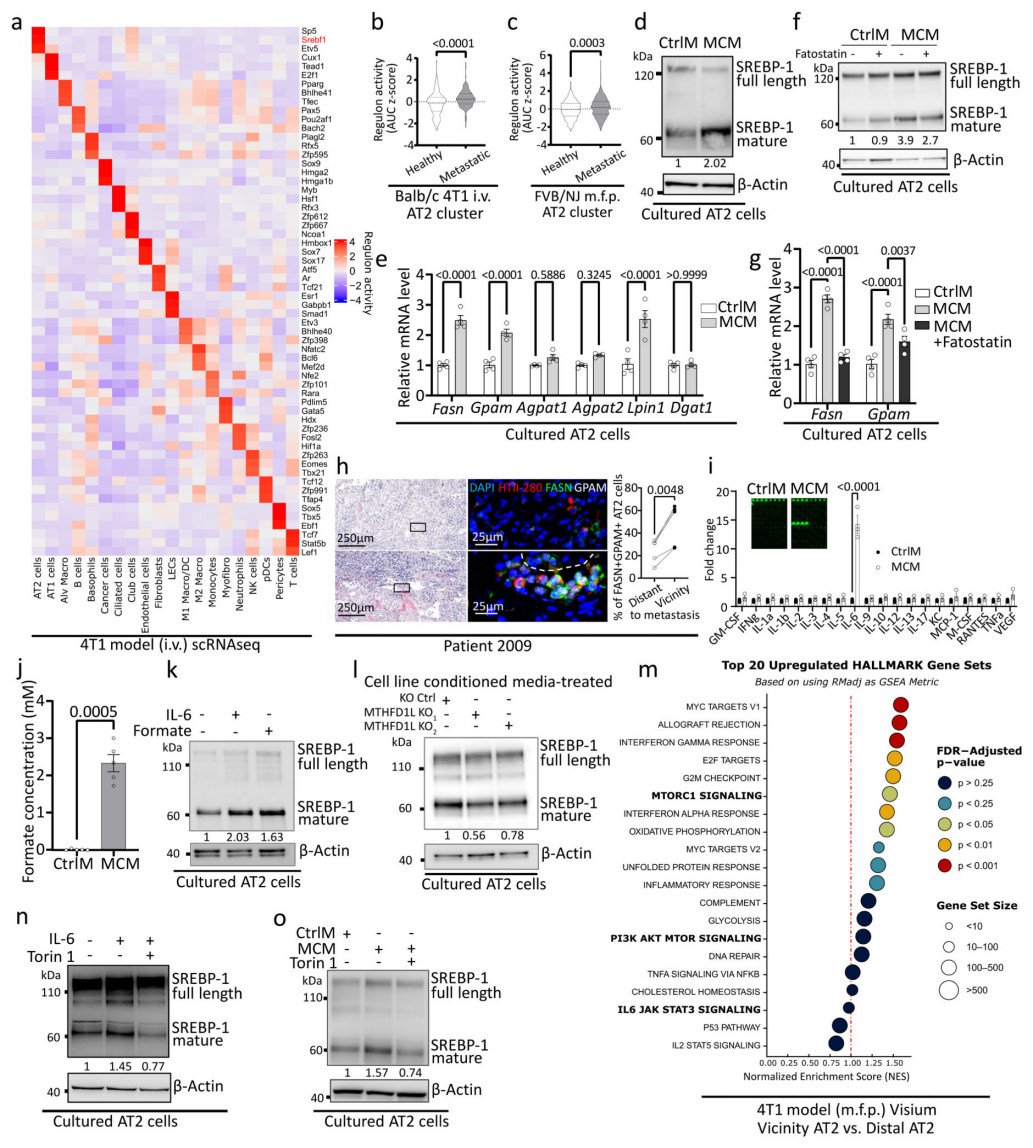
The metastasis secretome reprograms AT2 cell lipid metabolism by activating SREBP-1 a. Heatmap showing the top three most active regulons compared to all other cell types. Regulon activity (AUC scores) was calculated using AUCell implemented in SCENIC on scRNA-seq data from healthy and 4T1 i.v. injection-derived metastatic lungs. The average AUC score was calculated across all cells belonging to each cluster. The resulting matrix was z-score normalized per regulon across cell types. Each row represents a distinct regulon, and color intensity reflects its z-score normalized activity. b. *Srebp-1* activity in AT2 cell clusters from healthy lungs and 4T1 intravenous injection-derived metastatic lungs. Regulon activity was calculated by SCENIC algorithm and showing by z-score of area of under the curve. Statistical significance was determined using Mann-Whitney test. c. *Srebp-1* activity in AT2 cell clusters from healthy lungs and LM1 mammary fat pad injection-derived metastatic lungs. Regulon activity was calculated by SCENIC algorithm and showing by z-score of area of under the curve. Statistical significance was determined using Mann-Whitney test. d. Western blots of SREBP-1 and β-Actin in cell lysates extracted from control medium (CtrlM) and metastasis conditioned-medium (MCM) treated ex vivo cultured Balb/c mouse AT2 cells (n=3 biological replicates). Full length and cleaved mature SREBP-1 are marked in the blots. e. The changes in the expression of SREBP-1 potential target genes that are required for surfactant production upon the treatment of CtrlM or MCM on *ex vivo* cultured AT2 cells. The expression of genes is shown as relative fold change over CtrlM-treated cells. Data shown as mean ± SEM of four biological replicates, two-way ANOVA with Šídák multiple comparison test was performed to assess statistical significance. f. Western blots of SREBP-1 and β-Actin in cell lysates extracted from CtrlM or MCM combining with SREBP-1 inhibitor fatostatin (10μM) treated *ex vivo* cultured Balb/c mouse AT2 cells for 24 hours- (n=3 biological replicates). Full length and cleaved mature Srebp-1 are marked in the blots. g. The changes in the expression of *Fasn* and *Gpam* in *ex vivo* cultured AT2 cells treated with CtrlM, MCM or MCM combining with SREBP-1 inhibitor fatostatin (10μM) for 24 hours. The expression of genes is shown as relative fold change over CtrlM-treated cells. Data shown as mean ± SEM of four biological replicates, two-way ANOVA with Dunnett’s multiple comparison test was performed to assess statistical significance. h. Representative H&E staining and Opal multiplex IHC staining of AT2 marker HTII-280(red), FASN (green), GPAM (white) and DAPI (blue) in the lung tissue distant to metastasis and lung tissue in the vicinity of metastasis from breast cancer patient 2009 of the UPTIDER program. An approximate region corresponding to the Opal multiplex IHC staining is indicated on the H&E image, which was obtained from a serial section located several sections away. Scale bars: 250 μm (H&E) and 25 μm (IHC). n = 5 breast cancer patients (3 areas per patient per condition) were examined. The percentage of FASN and GPAM positive AT2 cells is shown on the right panel. Statistical significance was determined using paired t-test. i. The fold change of cytokine levels between CtrlM and MCM samples (n = 3 per condition) was analyzed using RayBio® Label-Based Mouse Antibody Arrays according to the manufacturer’s instructions. Representative images of one well from each condition are shown along with the corresponding quantification. j. Formate concentrations in CtrlM and MCM are measured by LC-MS. Concentrations are shown as mean ± SEM of five replicates from two independent batches. Statistical significance was determined using unpaired two-tailed t-test. k. Western blots of SREBP-1 and β-Actin in cell lysates extracted from recombinant IL-6 (2ng/mL) or sodium formate (500μM) treated *ex vivo* cultured Balb/c mouse AT2 cells for 24 hours (n=3 biological replicates). Full length and cleaved mature SREBP-1 are marked in the blots. l. Western blots of SREBP-1 and β-Actin in cell lysates extracted from 4T1 control (KO ctrl) and two independent 4T1-MTHFD1L KO (MTHFD1L KO_1_ and KO_2_) cell lines conditioned media treated *ex vivo* cultured Balb/c mouse AT2 cells (n=3 biological replicates). Full length and cleaved mature SREBP-1 are marked in the blots. m. GSEA-derived normalized enrichment scores (NES) for the top 20 up-regulated HALLMARK gene sets, determined from the Visium data in Figure 4a, comparing AT2 cell-enriched spots in metastasis-vicinity *vs* metastasis-distal regions. Dot colors and areas indicate FDR-adjusted *P*-values and gene-set sizes, respectively, with NES values shown in the x axis. Raw *P-*values were based on *fgsea*’s adaptive multilevel splitting Monte Carlo approach, and were subject to FDR adjustment using the Benjamini-Hochberg (BH) approach, considering the results for all 50 available HALLMARK gene sets (i.e. not only those for the 20 gene sets shown). n. Western blots of SREBP-1 and β-Actin in cell lysates extracted from recombinant IL-6 (2ng/mL), and from recombinant IL-6 (2ng/mL) combining with mTOR inhibitor Torin1 (20nM) treated *ex vivo* cultured Balb/c mouse AT2 cells for 24 hours (n=3 biological replicates). Full length and cleaved mature SREBP-1 are marked in the blots. o. Western blots of SREBP-1 and β-Actin in cell lysates extracted from CtrlM, MCM and MCM combining with mTOR inhibitor Torin1 (20nM) treated *ex vivo* cultured Balb/c mouse AT2 cells for 24 hours (n=3 biological replicates). Full length and cleaved mature SREBP-1 are marked in the blots.

Next, we asked whether lung metastases can reprogram the metabolism of AT2 cells to further boost lipid availability in their vicinity. To answer this, we first identified active transcription factors (TFs) in different lung-resident cell types, by applying single-cell regulatory network inference and clustering (SCENIC) (24) to scRNA-seq data from the lungs of healthy mice and mice injected i.v. with 4T1 cancer cells (22) or orthotopically injected with LM1 cells. Specifically, we focused on the top three most activated TFs across each annotated cell type (relative to all other cell types) in the TF regulon analysis (Figure 5a, Supplementary Figure 4a). One of the most active and widely expressed TFs in AT2 cells was sterol regulatory element-binding transcription factor 1 (gene name: *Srebf1*, protein name: SREBP-1) (Figure 5a, Supplementary Figure 4a-b), which is a master regulator of lipid metabolism (25). In line with this, the SCENIC analysis predicted an increased activity of SREBP-1 in the AT2 cells of metastatic lungs compared to those of healthy control lungs (Figure 5b-c).

To assess the influence of metastases on AT2 cells in their vicinity we treated *ex vivo* cultured AT2 cells with the secretome of the metastases. Specifically, we isolated AT2 cells from the lungs of healthy Balb/c mice (Supplementary Figure 4c) and cultured them in a Matrigel-based 3D system (26,27). After 8-12 days, the AT2 cells formed alveolar-like 3D structures (Supplementary Figure 4d) and expressed SFTPC, showing that they retained an AT2-cell state (Supplementary Figure 4e). We then generated metastasis conditioned medium (MCM) containing metastasis-secreted factors by culturing resected lung metastases in serum-free standard cell culture medium. Subsequently, we treated AT2 cells with a mix of AT2 maintenance medium and MCM *ex vivo*. We observed that MCM treatment significantly increased the level of cleaved (mature) SREBP-1 in AT2 cells (Figure 5d). In line, the gene expression levels of several SREBP-1 targets were upregulated in AT2 cells in response to MCM treatment (Figure 5e, Supplementary Figure 4f). These included *Fasn* and *Gpam*, which are required for surfactant production by AT2 cells. FASN catalyzes the synthesis of fatty acids from acetyl-CoA and malonyl-CoA and was highly expressed in AT2 cells in the vicinity of metastases (Supplementary Figure 4g). GPAM catalyzes the conversion of glycerol-3-phosphate which was increased in the vicinity of metastases (Supplementary Figure 4h), as well as acyl-CoAs to lysophosphatidic acid. Next, we treated ex vivo cultured AT2 cells with MCM and the SREBP-1 inhibitor fatostatin and observed that fatostatin blocked the MCM-induced SREBP-1 activation (Figure 5f) as well as expression of *Fasn* and *Gpam* (Figure 5g). Based on these data, we concluded that metastases promote, via their secretome, an increase in SREBP-1 activity and in the expression of its target genes in *ex vivo* cultured AT2 cells. Subsequently, we analyzed metastatic lung tissues from five patients with breast cancer enrolled in the postmortem tissue donation program UPTIDER (NCT04531696, KU/UZ Leuven) and assessed the expression of the SREBP-1 targets FASN and GPAM. Accordingly, we found that the fraction of both FASN-positive and GPAM-positive AT2 cells increased in the vicinity compared to the distance of the metastases in patients (Figure 5h). Thus, this provides evidence that SREBP-1 activity may increase in AT2 cells of metastatic lungs from patients and mice.

Next, we investigated potential mechanisms by which lung metastases upregulate SREBP-1 activity in AT2 cells. To address this question, we screened a panel of cytokines and metabolites in MCM compared to control media. For the metabolites, we focused on lipids, because their scarceness can stimulate SREBP-1 activity (28,29), as well as formate, which was previously linked to lipid metabolism in breast cancer cells (30). Total fatty acids, assessed by hydrolysis and subsequent fatty acid quantification, were either not changed or increased in MCM compared to control medium, suggesting that SREBP-1 activity was not triggered by a lack of lipids in the MCM (Supplementary Figure 4i). However, within the other measured molecules we found formate and interleukin 6 (IL-6) to be highly increased in MCM compared to control medium (Figure 5i-j). Consistently, the Visium spatial transcriptomics data showed that IL-6 signaling-related gene sets were upregulated in AT2 cell-enriched spots in the vicinity of metastases relative to distal regions, concomitant with an upregulation of *Fasn* expression (Supplementary Figure 4j-k). Therefore, we treated AT2 cells with recombinant IL-6 and with sodium formate and found that both were sufficient to increase the level of cleaved (mature) SREBP-1 in *ex vivo* cultured AT2 cells (Figure 5k). Next, we knocked out methylenetetrahydrofolate dehydrogenase (NADP+ dependent) 1 like (*Mthfd1l*), which encodes the enzyme catalyzing formate production (31) in 4T1 cells (Supplementary Figure 4l-m) and generated conditioned media from these cells. We found that the increase in cleaved (mature) SREBP-1 was attenuated in AT2 cells treated with conditioned media from *Mthfd1l* knockout 4T1 cells compared to control conditioned medium (Figure 5l). To target the effects of IL-6 on AT2 cells, we subsequently focused on the canonical Janus kinase (JAK) signaling axis, which can be activated downstream of IL-6 (32). Yet treatment of ex vivo cultured AT2 cells with IL-6 and the JAK inhibitor Fedratinib did not prevent the increase in cleaved (mature) SREBP-1 (Supplemental Figure 4n). Therefore, we next used the Visium data to identify enriched gene sets that could connect IL-6 to SREBP-1 activation. We observed that AT2 cell-enriched spots in the vicinity of metastases showed an enrichment of gene sets indicative of mTOR activity (Figure 5m), which is a known regulator of SREBP-1 (33) and can be activated by IL-6 (34). Thus, we treated ex vivo cultured AT2 cells with IL-6 or MCM, each combined with the mTOR inhibitor Torin1. We found that Torin1 treatment reduced the increase of cleaved (mature) SREBP-1 induced by IL-6 and MCM (Figure 5n-o). Taken together, these findings suggest that both IL-6 and formate, present in the metastasis secretome, contribute to the upregulation of SREBP-1 activity in ex vivo cultured AT2 cells.

### Targeting GPAM and FASN in AT2 cells impairs metastases growth

**Figure 6:**
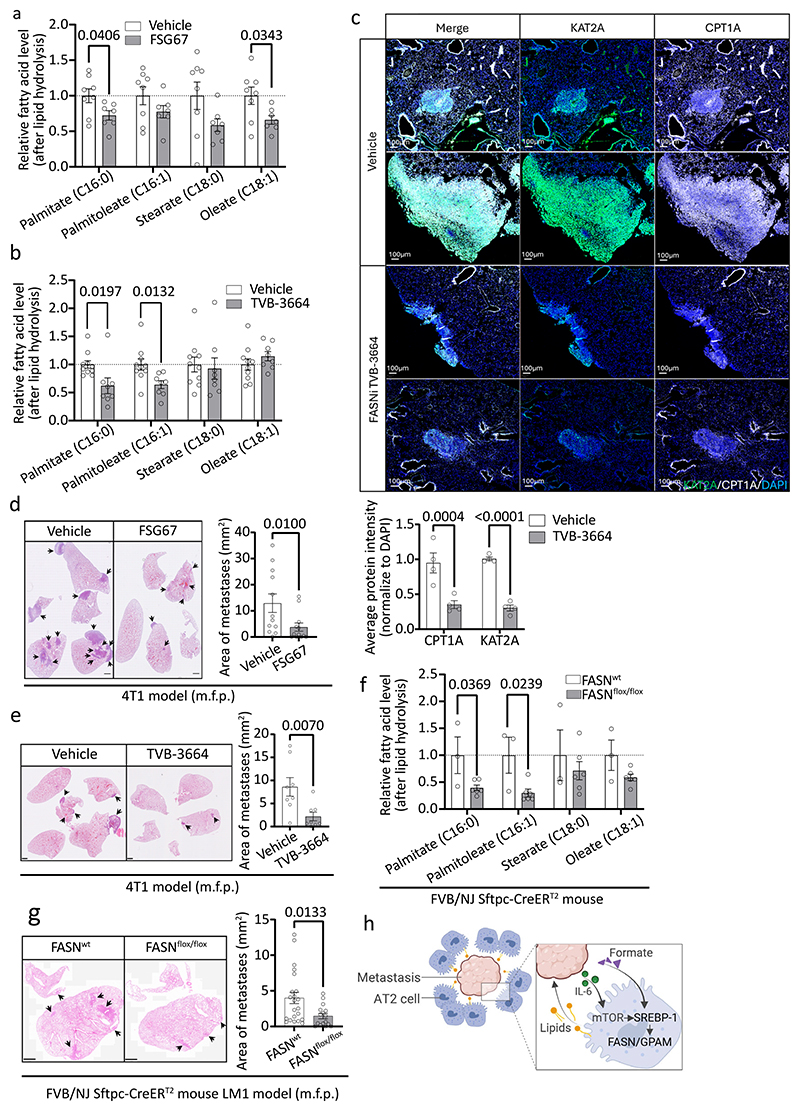
Targeting GPAM and FASN in AT2 cells impairs metastases growth a. Relative fatty acid abundance in the lung interstitial fluid of vehicle (n = 8) and Gpam inhibitor FSG67 treated (n = 7) mice. Data shown as mean ± SEM of biological replicates, unpaired two-tailed t-test was performed to assess statistical significance. b. Relative fatty acid abundance in the lung interstitial fluid of vehicle (n = 10) and Fasn inhibitor TVB-3664 treated (n = 8) mice. Data shown as mean ± SEM of biological replicates, unpaired two-tailed t-test was performed to assess statistical significance. c. Representative Opal multiplex IHC staining for KAT2A (green), CPT1A (white), and DAPI (blue) in metastatic lung tissues from vehicle- and FASN inhibitor TVB-3664–treated 4T1 m.f.p.–injected mice. Scale bars represent 100 μm. For quantification metastases were identified by H&E staining on the same tissue sections. KAT2A and CPT1A signals are normalized to DAPI signal and are presented as mean ± SEM of n=4 mice for each conditions. Two-way ANOVA with Šídák multiple comparison test was performed to assess statistical significance. d. Representative H&E staining of tissue from Balb/c mice injected with 4T1 cells into the mammary fat pads. Primary tumors were resected 7 days after mammary fat pad injection, and treatment with vehicle and FSG67 was initiated via daily intraperitoneal injection (i.p.) with a concentration of 5mg/kg body weight. Arrows indicate metastatic lesions. Scale bars represent 1mm. The metastatic area in the lungs of vehicle (*n* = 12 mice) and FSG67 (*n* = 12 mice) treated mice is presented as mean ± SEM, Mann-Whitney test was performed to assess statistical significance. e. Representative H&E staining of lung tissue from Balb/c mice injected with 4T1 cells into the mammary fat pads. Primary tumors were resected 7 days after mammary fat pad injection, and treatment with vehicle or TVB-3664 was initiated via daily oral gavage with a concentration of 3mg/kg body weight. Arrows indicate metastatic lesions. Scale bars represent 1mm. The metastatic area in the lungs of vehicle (*n* = 8 mice) and TVB-3664 (*n* = 8 mice) treated mice is presented as mean ± SEM, Mann-Whitney test was performed to assess statistical significance. f. Relative fatty acid abundance in the lung interstitial fluid of FASN wild type (*Sftpc-CreER*^*T2*^;FASN^wt/wt^, n = 3) and FASN AT2 conditional knockout (*Sftpc-CreER*^*T2*^; FASN^flox/flox^, n = 6) mice. Data shown as mean ± SEM of biological replicates, unpaired two-tailed t-test was performed to assess statistical significance. g. Representative H&E staining of lung tissue from FASN wild type and FASN AT2 conditional knockout mice injected with LM1 cells into mammary fat pads. Arrows indicate metastatic lesions. Scale bars represent 1mm. The metastatic area in the lungs of FASN wild type mice (*n* = 22 mice) and FASN AT2 conditional knockout mice (*n* = 16 mice) is shown as mean ± SEM, unpaired two-tailed t-test was performed to assess statistical significance. h. Established lung metastases secrete factors, including IL-6 and formate, that activate alveolar type II (AT2) cells to enhance surfactant lipid production by activating SREBP-1 signaling, which induces the expression of the SREBP-1 target genes *FASN* and *GPAM*. The enrichment of surfactant lipids further promotes metastasis development. (h. Created with BioRender.com)

Finally, we investigated whether metastases require the lipids produced by AT2 cells for effective growth. To impair the lipid production of AT2 cells we inhibited GPAM and FASN. Treatment with the GPAM inhibitor FSG67 (35) (5mg/kg body weight, daily i.p.) or the FASN inhibitor TVB-3664 (36) (3mg/kg body weight, daily oral gavage) did not change the lung morphology of mice (Supplementary Figure 5a-b). Yet, the abundance of palmitate- and palmitoleate-containing lipids decreased by approximately 28% and 35% in the lung interstitial fluid of mice treated with FSG67 or TVB-3664, respectively, compared to the vehicle-treated group, based on the quantification of fatty acids after lipid hydrolysis (Figure 6a-b). This finding was confirmed by lipidomics analysis of the lung interstitial fluid of mice treated with TVB-3664 or vehicle, showing that systemic inhibition of GPAM or FASN decreased lipid production (Supplementary Figure 5c).

Next, we determined whether the change in palmitate-containing lipid availability impacts the molecular state of cancer cells. We have previously shown that cancer cells respond to palmitate-containing lipids of their environment by increasing the expression of carnitine palmitoyltransferase 1A (CPT1A) and lysine acetyltransferase 2A (KAT2A), resulting in elevated palmitate oxidation, and in turn promoting metastases growth via protein acetylation (12). Therefore, we stained metastases from control and TVB-3664 treated mice for CPT1A and KAT2A. We found a significant decrease in the expression of these two proteins in the metastases from TVB-3664 treated mice (Figure 6c), providing evidence that the decrease in palmitate containing lipids provided by AT2 cells changes the molecular state of the cancer cells within the metastases.

Subsequently, we assessed metastasis growth in mice treated with GPAM and FASN inhibitors. We injected 4T1 cancer cells into the m.f.p. of mice, surgically removed the primary tumor after 7 days, treated the mice with FSG67 (5mg/kg body weight, daily i.p.) or TVB-3664 (3mg/kg body weight, daily oral gavage), and assessed the lung metastases at the endpoint. We observed that inhibiting GPAM with FSG67 and FASN with TVB-3664 reduced metastases growth by 71% and 75% respectively, compared to vehicle treatment (Figure 6d-e). Importantly, this reduction in lung metastases was associated with an effect of GPAM and FASN inhibition in the lung microenvironment and not a cancer cell-intrinsic effect, as genetic and pharmacologic inhibition of GPAM and FASN in breast cancer cells did not alter spheroid and lung metastases growth (Supplementary Figure 5d-g, Supplementary Figure 6a-h).

Finally, we investigated whether impairing the lipid production specifically in AT2 cells is sufficient to reduce lung metastasis growth. To do so, we targeted *Fasn* expression specifically in AT2 cells. We generated *Sftpc-CreER*^*T2*^; FASN^flox/flox^ mice to delete *Fasn* specifically in AT2 cells upon tamoxifen treatment (Supplementary Figure 6i). Lungs from *Sftpc-CreER*^*T2*^; FASN^wt/wt^ littermates treated with tamoxifen were used as controls. We investigated the effect of *Fasn* deletion specifically in AT2 cells by measuring the abundance of fatty acids derived from all lipids by hydrolysis in lung interstitial fluid using mass spectrometry. We observed that *Sftpc-CreER*^*T2*^; FASN^flox/flox^ mice exhibited a more than 60% decrease in total palmitate and palmitoleate containing lipids in their lung interstitial fluid compared to *Sftpc-CreER*^*T2*^; FASN^wt/wt^ control mice (Figure 6f). Subsequently, we injected LM1 cancer cells into the m.f.p. of *Sftpc-CreER*^*T2*^; FASN^flox/flox^ and *Sftpc-CreER*^*T2*^; FASN^wt/wt^ mice pre-treated with tamoxifen (6 days after tamoxifen treatment) and assessed metastases growth after 28 days. Strikingly, deletion of *Fasn* only in AT2 cells was sufficient to reduce metastases growth by 64% compared to control mice (Figure 6g). Take together these findings show that targeting GPAM and FASN systemically and AT2 cell-specifically decreases the lipid availability in the lung environment resulting in impaired lung metastasis growth.

In conclusion, we discovered that metastases activate AT2 cell proliferation, and lipid release through activation of the transcription factor SREBP-1 which fosters metastatic growth, and that inhibiting the SREBP-1 targets FASN and GPAM in AT2 cells is sufficient to reduce metastasis growth (Figure 6h).

## Discussion

Here, we discovered that breast cancer-derived lung metastases reprogram AT2 cells in their vicinity to exploit them as lipid feeder cells. Furthermore, our findings identify SREBP-1 as a key transcriptional regulator driving this reprogramming in AT2 cells, and FASN as well as GPAM as drug targets in AT2 cells reducing metastasis growth.

AT2 cells have been reported to contribute to the formation of the pre-metastatic niche by responding to signals derived from the primary tumor (12,37). Complementary to these findings, our study uncovers a progressive enrichment of AT2 cells and lipids in the vicinity of lung metastases, independent of the presence of primary tumors. This finding is clinically highly relevant because most metastatic breast cancer patients have faced the metastases diagnosis months to years after primary tumor resection (38). Moreover, we identify AT2 cells as lipid feeder cells to overt metastases. This spatiotemporal enrichment of AT2 cells during metastasis growth is complementary to a recent study which observed the migration of AT2 cells between alveolar units to support alveolar repair in response to lung injury (39). Notably, AT2 cells have been considered the origin of lung adenocarcinoma (40,41). This raises the question whether the lipid metabolism of AT2 cells also contributes to lung cancer formation and progression. While the direct connection between AT2 cell lipid metabolism and lung cancer formation remains to be determined, it is tempting to speculate that it may play a role in supporting tumorigenesis in primary lung cancers. Investigating this potential link could provide new insights into the role of AT2 cells in lung cancer development and highlight additional therapeutic opportunities.

SREBP-1 and its downstream targets FASN and GPAM have been extensively studied in cancer cells (42). Thereby it was found that targeting SREBP-1, FASN and GPAM in cancer cells reduced the growth of liver cancer, lung cancer and leukemia, respectively (43–45). This has led to the development of a FASN inhibitor TVB-2640, which is currently investigated in clinical trials for non-small-cell lung carcinoma with KRAS mutation (NCT03808558) and advanced breast cancer (NCT03179904) (46). Moreover, there are ongoing efforts to target the lipid uptake of cancer cells (47,48) and immune cells (49). We add to this cancer cell intrinsic and immune cell knowledge a key role of lung resident cells by showing that the presence of metastases reprograms AT2 cells into lipid feeder cells, mediated in part by IL-6 and formate of the metastasis microenvironment that stimulate the activity of SREBP-1. In turn, AT2 cells in the vicinity of metastases release higher amounts of surfactant lipids with a profile similar to that of normal lung. In line with this, targeting the lipid metabolism of AT2 cells is sufficient to decrease metastasis growth. This highlights the importance of metabolic communications between cancer cells and organ resident cells as well as the importance of organ resident cells, and particularly AT2 cells, as drug targets in metastasis therapy.

Multiple pre-clinical studies have shown the potential of targeting cancer metabolism to inhibit tumor progression (50) and several metabolic inhibitors targeting cancer cells or immune cells have been or currently are in clinical trials (NCT03808558, NCT04471415, NCT03272256, NCT02746081, NCT03179904). We complement these current efforts by using spatial metabolomics platforms to identify that metastatic tissues display a heterogeneity of metabolism that stems not only from the cancer cells and immune cells of the tumor microenvironment, but also from organ resident cells. Thus, this work may foster the use of spatial metabolomics data to identify novel therapeutic strategies against cancer metastasis and beyond.

## Methods

### Cell culture

4T1 (CRL-2539, RRID: CVCL_0125) and HEK293T (CRL-3216, RRID: CVCL_0063) cell lines were purchased from American Type Culture Collection (ATCC). The EMT6.5 cell line was provided by R. Anderson, E0771-Met clone by Prof. Mazzone. LM1 cell line was established in order to study lung metastasis from mammary gland tumors, in an MMTV-Myc setting. Specifically, a cell line (“Myc310”) was established following multiple (>20) *in vitro* passages of cells isolated from a transgenic MMTV-Myc tumor. LM1 cell line was established by isolating the lung metastases from Myc310 mammary gland tumor-bearing mice, and expanding the tumor cells *in vitro*. Only metastatic cells remained in culture after two weeks, and their identity was confirmed by immunofluorescence staining (Myc^+^ and CK8^+^) (Supplementary Figure 2a). The 4T1, EMT6.5 and E0771-Met cells were grown in Roswell Park Memorial Institute (RPMI) 1640 medium and HEK293T were grown in Dulbecco’s modified Eagle medium (DMEM), both supplemented with 10% FBS, 1% penicillin (50 U/mL) and 1% streptomycin (50 μg/mL). LM1 cells were grown in MMEC media (DMEM/F12, 10% FBS, 10mM HEPES, 2 mM glutamine, 5ug/mL insulin, 1 ug/mL hydrocortisone, 10 ng/mL EGF, 100 ug/mL penicillin/streptomycin). All cells were maintained at 37 °C, 5% CO2 and 95% relative humidity and screened to be mycoplasma free via routine testing (monthly) with the Mycoalert detection kit (Lonza). For 3D growth conditions, plates were covered with soft agar mixed 1:1 with culture media as described (51). Specific number of cells were plated on top of the base agar and incubated for 3–5 days. Representative pictures of 3D spheroids were taken at the end of the experiment (3-5 days) with the Motic Images Plus 2.0 software (Motic) and were quantified using Image Studio Lite 5.2.

### RNA extraction and gene expression analysis by reverse transcription-quantitative PCR (RT-qPCR)

Total RNA from frozen tissues or cell lines was isolated using TRIzol Reagent (Thermo Fisher Scientific) and the isolated RNA was quantified using the NanoDrop One Microvolume UV-Vias spectrophotometer (Thermo Fisher Scientific). The complementary DNA synthesis was performed using qScript cDNA Synthesis kit (Quanta) according to the manufacturer’s protocols. RT-qPCR was performed using SYBR Green PCR Master Mix, Low ROX (Quantabio) on the 7500 Fast Real Time PCR System (Applied Biosystems, Life Technologies). The relative mRNA expression level was normalized to an endogenous housekeeping gene and relative expression was analyzed using the 2^−ΔΔCt^ method. Gene-specific primers are described in Supplementary Table 1.

### Generation of knockdown cell line

*Gpam* and *Fasn* knockdown cell lines were generated using the lentiviral pLKO-shRNA1.5 vector expressing short hairpin RNAs against Gpam (purchased from the Belgian Coordinated Collections of Microorganisms) or *Fasn* (purchased from Merk Sigma-Aldrich), detailed information is listed in Supplementary Table 2. The pLKO.1-puro non-target shRNA control plasmid (Cat# SHC016) expressing a non-targeting shRNA sequence was used as control. Briefly, lentiviral particles were produced in HEK293 cells. Transduction of cells was performed overnight and the media was replaced the next day. Polyclonal cells were selected with puromycin, 1 μg/mL for EMT6.5, 2 μg/mL for 4T1 and E0771-Met cells, and 5 μg/mL for LM1 cells. The knockdown was confirmed by RT-qPCR, and a list of qPCR primers is described in Supplementary Table 2.

### Mouse lung tissue dissociation

6–8-week-old BALB/c mice were anesthetized with ketamine (100 mg/kg) and xylazine (10 mg/kg) and lungs were perfused through the right ventricle (52), extracted and washed in blood bank saline. Tissues were then dried and minced with blades and subsequently incubated with a solution of 0.3 mg/mL liberase (Roche) and DNase1 (1 µg/mL) in 2 mL of RPMI at 37 °C for 45 minutes with occasional vortexing. The dissociated lungs were quenched with 3% fetal bovine serum in PBS supplemented with 2mM EDTA, filtered through a 70 µm cell strainer and centrifuged for 5 minutes at 300 x g. The cell pellet was washed, incubated with Red Blood Cell Lysis buffer (Merck) and strained with a 40 µm cell strainer. The dissociated lung single-cell suspension was kept on ice until downstream analyses.

### Primary alveolar type II cell isolation by flow cytometry and *in vitro* culture/treatment

Mouse primary alveolar type II cells were isolated by flow cytometry sorting following the gating strategy previously reported (53). Briefly, the dissociated healthy mouse lung tissue was counted as described above and incubated at 4 °C for 10 minutes with anti-mouse CD16/CD32 (Fc block, BD Biosciences, Cat# 553142, RRID: AB_394656). The samples were then stained at 4 °C for 20 minutes with fluorophore-conjugated antibodies against CD45 (BD Bioscience, Cat# 550994, RRID: AB_394003, 1:200 dilution), CD31 (BD Bioscience, Cat# 562939, RRID: AB_2665476, 1:200 dilution), CD49f (Thermo Fisher Scientific, Cat# 25-0495-82, 1:100 dilution), EpCAM (Thermo Fisher Scientific, Cat# 17-5791-82, RRID: AB_2716944, 1:200 dilution), MHC-II (BioLegend, Cat# 107643, RRID: AB_2565976, 1:100 dilution) and Viability efluor780 (Thermo Fisher Scientific, Cat# 65-0865-14, 1:800 dilution). Sorting was performed using a BD FACSAria™ Fusion Flow Cytometer. Mouse alveolosphere culture, using mouse AT2 maintenance media (AMM), was perform as described previously (26,27). Briefly, sorted AT2 cells (5 × 10^3^) were resuspended in AMM and mixed with an equal volume of growth factor-reduced Matrigel (Corning, Cat# 356231, Lot#3108001). A drop of 50μL of cells-media/Matrigel mixture were plated in each well of a 12-well plate. After seeding, the plates with droplets were incubated at 37°C, and after 20 minutes, 1mL AMM was added to each well. The media was changed every other day. The representative pictures of 8 day and 12 day-cultured AT2 cells were taken using Motic Images Plus 2.0 software (Supplementary Figure 4d). The *in vitro* treatments, including CtrlM/MCM, Fatostatin (MedChem Express, Cat# HY-14452), Torin1 (Selleckchem, Cat# S2827), Fedratinib (MedChem Express, Cat# HY-10409), recombinant mouse IL-6 (Biolegend, Cat# 575702), and sodium formate (Merck Sigma-Aldrich, Cat# 71539) under specified conditions, were initiated after 10-12 days of culturing, when the 3D structure had formed.

### *In vitro* 3D spheroid growth assays

To measure 3D spheroid growth, cells were plated on top of a soft agar mixture (51) of 50% agar and 50% cell culture media. 4T1 and E0771-Met cells with different knock down conditions, were cultured in six-well plates upon specified conditions for 5 days and representative pictures of each well were taken using Motic Images Plus 2.0 software (Motic). Spheroid area was analyzed using Image Studio Lite 5.2 with at least five representative pictures per experimental condition.

### *In vivo* mouse experiments

All animal experiments complied with ethical regulations and were approved by the Institutional Animal Care and Research Advisory Committee of KU Leuven (ECD nos. P007/2020, P048/2020 and P025/2020). The work involving the MMTV-Myc and MMTV-PyMT mouse models, as well as the models derived by Myc310 and LM1 cell lines, was performed in accordance with United Kingdom Home Office regulations under the Animals (Scientific Procedures) Act 1986 (Project licence P609116C5). This work was also approved by the Francis Crick Animal Welfare and Ethical Review Body. Mice were housed under a regimen of 12 h light-12 h dark in non-SPF (conventional) facility with a constant supply of food and water. Sample size was determined using power calculations with *B* = 0.8 and *P* < 0.05 based on preliminary data and in compliance with the 3R system: replacement, reduction, refinement. All mice were randomized before injections and samples were analyzed in a blinded manner.

For lung metastasis models, 6–8-week-old female Balb/c (for 4T1 cells) or 8-10-week old FVB/NJ (for LM1 cells) mice were inoculated with 4T1 (1 × 10^6^) or LM1 (5 × 10^4^) cells in the mammary fat pad (m.f.p.), or injected intravenously (i.v.) with 4T1 (1 × 10^5^) or EMT6.5 (1 × 10^5^) cells. For 4T1 cell injections, mice were euthanized after 21-23 days for m.f.p. or 13-17 days for i.v. injections with an intraperitoneal (i.p.) injection of 10 µL/g containing ketamine (100 mg/kg)-xylazine (10 mg/kg) or 50 µL of a 60 mg/mL Dolethal (pentobarbital sodium) solution (Vetoquinol). For LM1 cell injections (m.f.p.) mice were euthanized at 21-28 days post-injection by cervical dislocation in accordance with Schedule 1 of the Animals (Scientific Procedures) Act 1986 (UK).

For *in vivo* treatment with the FASN inhibitor, TVB-3664 (MedChemExpress, Cat# HY-120062) and the GPAM inhibitor, FSG67 (Focus Biomolecules, Cat# 10-4577), 4T1 cells (1 × 10^6^) were injected into m.f.p. of 6–8-week-old female Balb/c. After 7 days, the primary tumors were resected, and the treatments were initiated. The FASN inhibitor, TVB-3664 (3mg/kg), was administered daily by oral gavage as an emulsion in 30% Polyethylene glycol 400 (Sigma) in water. The GPAM inhibitor, FSG67 (5mg/kg) was administered daily by i.p. injection in PBS. The corresponding vehicles were administered using the same methods. Mice were euthanized after 23-26 days post-injection for metastatic burden evaluation by histological staining.

To specifically knock out FASN on lung AT2 cells, *Sftpc-CreER*^*T2*^ (54) mice (C57BL/6 background, JAX stock #028054, RRID:IMSR_JAX:028054) were crossed for 10 generations into FVB/NJ background, following the cross with FASN^flox/flox^ line (55) to generate *Sftpc-CreER*^*T2*^;FASN^flox/flox^ mice. The breeding was designed to produce both *Sftpc-CreER*^*T2*^;FASN^flox/flox^ and *Sftpc-CreER*^*T2*^;FASN^wt/wt^ genotypes in the same litter. *Sftpc-CreER*^*T2*^;FASN^wt/wt^ mice were used as a control. To induce Cre activity specifically in AT2 cells, the *Sftpc-CreER*^*T2*^;FASN^flox/flox^ and *Sftpc-CreER*^*T2*^;FASN^wt/wt^ female mice, aged between 8-10 weeks, were administered 200mg/kg of body weight tamoxifen (i.p., Stock: 20mg/mL tamoxifen (Sigma) in olive oil) on day 1 and day 3 (56). Six days after the second tamoxifen injection, the efficiency of Cre activity was confirmed by evaluating FASN expression by immunofluorescence staining in SFTPC-positive AT2 cells (Supplementary Figure 6i). Six days after the second tamoxifen injection, LM1 (5 × 10^4^) cells were injected into mouse m.f.p., and 28 days later, primary tumors and lungs were collected for further analyses. For all experiments, mice were monitored for detection of humane endpoints, determined using a score sheet (tumor size of 1.8 cm^3^, loss of ability to ambulate, labored respiration, surgical infection or weight loss over 20% of the initial body weight).

### Metastasis conditioned-media generation

The procedure to generate metastasis conditioned-media (MCM) is modified on the previously described tumor condition media generation (12). Briefly, 2×10^5^ 4T1 cells were injected into 6 week-old Balb/c mice (i.v.). At 12 days post-injection, the mice were sacrificed and lung metastases were picked under microscope. Lung metastases were cut into small pieces and cultured at 37 °C in certain amount of DMEM (no FBS) + 1% penicillin and 1% streptomycin (normalized by the weight of metastases 15mL/g). The same amount of media without metastasis was incubated in parallel to generate control medium (CtrlM). After 72 h, media was transferred to a 70 μm cell strainer and spun down for 10 min at 1,000g. Supernatants from three different tumors were pooled together to cover the variability between tumors. HEPES (20 mM) was added to the media and this was filtered and stored at 4 °C no more than 2 weeks after collection. For long-term storage, CtrlM and MCM were stored at -80 °C.

### CRISPR-Clone generation

MTHFD1L-KO were generated using following CRISPR-Cas9 plasmids constructed by VectorBuilder (MTHFD1L: VB241115-1113pwx, Scramble: VB220214-1067vbu). 4T1 wild type cells were reverse transfected with the plasmids using Lipofectamin3000 according to manufactures instruction. After 2 days of incubation cells were detached, stained with ZombieNIR according to manufactures instruction and ZombieNIR^-^ mCherry^+^ cells were single sorted into 1 cell/well in a 96-well plate. After 10 days of incubation and regular media replenishment, single clones were picked and further analyses.

### GC-MS: Formate secretion-rate measurements

Formate secretion-rate measurements on 4T1 wild type, MTHFD1L KO control and two independent MTHFD1L KO cell lines were performed using GC-MS, following MCF derivatization of culture media supernatants, as described previously (31). In brief, cells were grown for 24h in culture media depleted of serine and supplemented with 400μM uniformly 13-C labelled Serine (Cambridge Isotope Laboratories, Cat# CLM-1574). An equal number of culture wells containing the same media but no cells (cell-free media) were incubated alongside the cells. Medium samples were mixed with internal standard (IS) sodium ^13^C,^2^H-formate (M^+2^) (final conc: 250 μmol/L; Sigma-Aldrich, CAS 1215684-17-5) prior to derivatization. All samples were handled in technical replicates including plastic blanks (LC-MS grade H_2_O, to account for background formate levels coming from solvents and/or plastics), cell-spent supernatants, and cell-free supernatants. GC-MS analysis was performed using an Agilent 7890 A GC coupled to an Agilent 5975 C inert XL Mass Selective Detector (Agilent Technologies). A sample volume of 1 μL was injected into a Split/Splitless inlet, operating in split mode (20:1) at 270 °C. The gas chromatograph was equipped with a 30 m (I.D. 250 μm, film 0.25 μm) DB-5MS capillary column (Agilent J&W GC Column, 122-5532 G). Helium was used as carrier gas with a constant flow rate of 1.4 ml/min. GC oven temperature was held at 80 °C for 1 min and increased to 130 °C at 10 °C/min followed by a post run time of 4 min at 280 °C. Total run time was 15 min. Transfer line temperature was set to 280 °C. Mass selective detector (MSD) was operating under electron ionization at 70 eV. MS source was held at 230 °C and the quadrupole at 150 °C. For precise quantification, measurements were performed in selected ion monitoring mode. Target ions (m/z) and dwell times are listed below: Benzyl formate (Formic acid) – M0, Quant-Ion 136.1 (m/z), dwell time 40 (ms); Benzyl formate (Formic acid) – M1, Quant-Ion 137.1 (m/z), dwell time 40 (ms); IS Benzyl formate (Formic acid ^13^C, D) – M0, Quant-Ion 138.1 (m/z), dwell time 40 (ms); IS Benzyl formate (Formic acid ^13^C, D) – M1, Quant-Ion 139.1 (m/z), dwell time 40 (ms); IS Benzyl formate (Formic acid ^13^C, D) – M2, Quant-Ion 140.1 (m/z), dwell time 40 (ms). GC-MS chromatograms were processed using Agilent MassHunter Quantitative Analysis for GC-MS, Version B.08.00. Formate secretion rates were calculated according to (31) using the formate_CORE script published on GitLab repository (https://git.lih.lu/vvoorsluijs/formate_core).

### Cytokine array

Mouse Cytokine Array (RayBiotech, Cat# GSM-CYT-1) was used to measure a panel of 20 mouse cytokines. CtrlM and MCM samples were hybridized according to the manufacturer’s instructions and scanned using a Typhoon™ FLA 9500 biomolecular imager (GE Healthcare) for glass slides. The scanning signals were extracted using ImageQuant TL software (version 11.0.1086.0) and were normalized by RayBiotech analysis tool.

### Hematoxylin and eosin (H&E) staining

H&E staining of lungs was performed to identify the morphology of lungs after drug treatment and the lung metastatic lesions. Fixed lung samples were embedded in paraffin and sliced sections of 7 µm for H&E staining. H&E images were scanned on an Axioscan 7 Microscope Slide Scanner (Zeiss) or AKOYA PhenoImager HT slide scanner (20x or 40x). Scanned slides were analyzed using QuPath software (v0.4.4) (57), and metastatic burden was determined by analyzing the area of metastases. All samples were analyzed blinded.

### Immunohistochemical staining of human and mouse lungs

Immunohistochemical (IHC) staining for SFTPC, KAT2A and CPT1A of mouse tissues was performed on formalin-fixed paraffin-embedded (FFPE) lung samples. In details, 7 µm thick slices of tissue were cut and mounted on BOND Plus Slides (Leica). Slides were dried for 16h at 37 °C and then stored at RT until experiment. On the day of experiment tissue was deparaffinized using Leica Autostainer XL (ST5010) followed by antigen retrieval with AR9 (Leica). Tissues were then blocked with blocking buffer (10% goat serum, Invitrogen, 0.5% BSA in TBS Buffer) for 30 min. The primary antibody SFTPC (Abcam, Cat# ab211326, RRID: AB_2927746,1:500 dilution), KAT2A (also known as GCN5L2, Cell Signaling, Cat# 3305, RRID:AB_2128281, 1:200 dilution), CPT1A (Cell Signaling, Cat#97361, RRID:AB_2895298, 1:100 dilution) was incubated for 1h at room temperature and followed by washes with PBS-T (PBS, 0.05% Tween-20). The secondary HRP (Dako Envision+ Single Reagents, HRP, rabbit, K4003) were incubated for 10 min. After washes with PBS-T, the OPAL dye was incubated for 10 min. Heat-mediated antibody stripping was performed to remove primary and secondary antibodies, allowing for additional rounds of labeling with other primary antibodies and DAPI (Spectral DAPI Akoya) staining. After the staining slides were mounted with antifade diamond mounting media (Invitrogen) and imaged using AKOYA PhenoImager HT slide scanner. For mouse lung tissues, after IHC staining and scanning, the coverslips were removed and the sections were washed twice with PBS-T. We then performed H&E staining and scanning on the same sections following the protocol described above.

IHC staining for SFTPC and PanCK of early and late metastatic mouse lung samples from PDX models was performed on FFPE samples. The sample preparation was described by previous study (15,16). The FFPE lung samples were sectioned at 4 µm. Following the same protocol used above with the details: antigen retrieval with 1x citrate buffer pH 6.0 (Sigma-Aldrich C9999), primary antibody PanCK (Dako, Cat# M3515, RRID: AB_2132885, 1:100 dilution), SFTPC (Abcam, Cat# ab211326, RRID: AB_2927746, 1:500 dilution), secondary HRP (AKOYA, Opal Polymer HRP Ms + Rb for PanCK) and HRP antibody (Dako Envision+ Single Reagents, HRP, rabbit, K4003 for SFTPC).

IHC staining for HTII-280, PanCK, FASN and GPAM of human patient tissues was performed on FFPE embedded lung samples. Clinicopathological information for every patient in the UPTIDER dataset is shown in Supplementary Figure 1a. The FFPE samples were sectioned at 7 µm. All the staining steps were performed with the BOND RX Fully Automated Research Stainer 21.2821 (Leica) and staining was done with OPAL 6 plex detection kit (Akoya NEL821001) according to manufacturer’s instructions. Antigen retrieval was performed with AR6 (Leica), and then blocked with blocking buffer (10% goat serum, Invitrogen, 0.5% BSA in TBS Buffer). The following primary antibodies were used: PanCK (Dako, Cat# M3515, RRID: AB_2132885, 1:200 dilution), Anti-HT2-280 antibody (Terrace Biotech, Cat# TB-27AHT2-280, RRID: AB_2832931, 1:150 dilution), Anti-FASN (Cell Signaling, Cat #3180, RRID: AB_2100796, 1:200diluition) and Anti-GPAM (Invitrogen, Cat# PA5-20524, RRID: AB_11155813, 1:500 dilution). As human metastatic tissues are precious human material some of the HT2-280 staining was done in parallel with other antibodies not relevant to this study (22). It was tested that the other antibodies did not interfere with HT2-280 staining. HT2-280 staining was visualized with OPAL 620 or 520, PanCK with OPAL780, FASN with OPAL570, and GPAM with OPAL690. After the staining slides were removed from the autostainer and mounted with antifade diamond mounting media (Invitrogen) and imaged using AKOYA PhenoImager HT slide scanner. Spectral unmixing was performed online with standard library provided by AKOYA Version 2.0 onwards and unmixed images were analysed using QuPath Version 0.5 onwards.

To quantify the number of AT2 cells, a PanCK-positive region was selected using the wizard tool and expended using the “Expand annotations” tool in QuPath to a 100-200 μm area surrounding the metastasis. For the quantification of AT2 cells in the lung distant to metastatic regions, the regions distant to the metastatic areas were randomly selected, ensuring no visible metastases were present within 250 μm of selected region, as determined by PanCK staining. In identified regions, cells were detected using DAPI with Qupath positive cell detection algorithm and the number of HTII-280-positive and HTII-280-negative cells were then calculated. For quantification of GPAM and FASN signals in the AT2 cells, three regions in the metastasis vicinity and three distant regions per patient were selected based on PanCK staining. Following this, nuclei in these regions were detected using the nuclei detection tool in QuPath. Threshold for AT2 positive and negative cells were defined, and AT2 cells were identified using high HTII-280 staining. Following this, the staining intensities of FASN and GPAM in each identified AT2 cell were extracted. For each patient, FASN and GPAM intensities were averaged across the three regions and compared.

### In vivo 5-bromo-2’-deoxyuridine (BrdU) labeling assay

To assess the proliferation of AT2 cells in metastatic lungs, the mice from 4T1 (m.f.p. and i.v.) lung metastatic mouse models were i.p. injected with two doses of BrdU in saline (Merck Sigma-Aldrich, B5002; 50 mg/kg) 30 hours and 6 hours prior to euthanasia. Animals were checked regularly for signs of discomfort (hunched back, shivering, low mobility) after the injection. Lung tissues were collected and fixed in 10% neutral-buffered formalin, paraffin-embedded, and sectioned at 7 µm. Slides were dried for 16h at 37 °C and then stored at RT until experiments.

BrdU incorporation was assessed by immunofluorescence staining. Tissues were blocked with blocking buffer (10% donkey serum, Merck Sigma-Aldrich Cat# S30, 1% BSA in TBS Buffer) for 1 hour at room temperature. Slides were then stained with primary antibody SFTPC (Abcam, Cat# ab211326, RRID: AB_2927746, 1:500 dilution) and Anti-BrdU antibody (Abcam, Cat# ab6326, RRID: AB_305426, 1:200 dilution) in blocking buffer, overnight at 4 °C. Slides were then washed and stained with an AlexaFluor 488 conjugated donkey anti-rabbit IgG (H+L) secondary antibody (Thermo Fisher Scientific, Cat# A-21206, RRID: AB_2535792, 1:400 dilution) and an AlexaFluor 647 conjugated donkey anti-rat IgG (H+L) secondary antibody (Thermo Fisher Scientific, Cat# A78947, RRID: AB_2910635, 1:400 dilution) in blocking buffer for 2 hours at room temperature. The slides were then washed again and followed by DAPI staining and mounted using ProLong Gold Antifade Mountant (Thermo Fisher Scientific, P36930). After drying overnight, the slides were imaged using AKOYA PhenoImager HT slide scanner. To quantify the number of BrdU^+^ AT2 cells in metastasis proximal and distal areas, the metastatic regions were identified based on H&E staining of the same tissue sections. Areas within 100-200 μm surrounding the metastases were identified as the metastasis vicinity, while regions distant from the metastatic areas were randomly seleted, ensuring that no visible metastases were present within 250 μm of selected regions. In these identified regions, cells were detected using DAPI with QuPath positive cell detection algorithm, and the percentage of BrdU- and SFTPC-positive cells among total SFTPC-positive cells was then calculated.

### Immunofluorescence staining of mouse lungs and *in vitro* 3D cultured AT2 cells

Fresh-frozen lung tissues or OCT embedded 4% PFA fixed 3D cultured AT2 cells were sectioned using a Leica CM3050s cryostat (10 µm thickness) and fixed in 4% paraformaldehyde (PFA; Invitrogen, 22B085301) for 10 minutes at room temperature. PFA was washed off with PBS-T and slides were incubated in blocking buffer (PBS-T, 1% BSA, 5% normal goat serum) for 30 minutes at room temperature. Slides were then stained with primary anti-mouse FASN antibody (Cell Signaling Technology, Cat# 3180, RRID: AB_2100796, 1:100 dilution) in blocking buffer, overnight at 4 °C. Slides were then washed and stained with an AlexaFluor-488 conjugated goat anti-rabbit IgG (H+L) secondary antibody (Thermo Fisher Scientific, Cat# A-11008, RRID: AB_143165, 1:300 dilution) in blocking buffer for 2 hours at room temperature. Since both primary antibodies were established in rabbit, for co-staining, the slides were then blocked in rabbit blocking buffer (PBS-T, 1% BSA, 5% normal rabbit serum) for 1 hour at room temperature, to block open binding arms of the secondary anti-rabbit antibody. Primary anti-mouse SFTPC antibody (Abcam, Cat# ab90716, RRID:AB_10674024) was conjugated to an AlexaFluor-555 fluorophore using the AlexaFluor-555 Lightning-Link® Conjugation Kit (Abcam, ab269820) following the manufacturer’s instructions. Slides were then incubated with the conjugated primary anti-SFTPC antibody (1:100 dilution in rabbit blocking buffer) for 2 hours at room temperature. Slides were washed in PBS-T, and DAPI solution was then added for 10 minutes to stain nuclei. Slides were washed in PBS once and mounted using ProLong Gold Antifade Mountant (Thermo Fisher Scientific, P36930). To quantify regional differences in the proportion of AT2 cells, metastatic regions were first identified using consecutive H&E staining. Areas of metastasis vicinity were defined as distance within 200 μm, while areas distant to metastasis were located at least 500 µm away from any metastasis. In identified regions, cells were detected using the DAPI signal, and AT2 cells were identified by SFTPC^+^ staining.

For the slides containing 3D cultured AT2 cells, the blocking buffer is PBS-T, 1% BSA, 5% donkey serum; the primary antibody is anti-SFTPC antibody (Abcam, Cat# ab211326, RRID: AB_2927746, 1:500 dilution) and the secondary antibody is an Donkey anti-Rabbit AlexaFluor-555 (Thermo Fisher Scientific, Cat# A-31572, RRID:AB_162543, 1:300 dilution). The slides were then washed and followed by DAPI staining and scanning.

Immunofluorescence staining for SFTPC, MUC1, LAMP3, and Ki67 of mouse lung tissues were performed on FFPE samples. Following the same protocol above with the details: blocking buffer (10% goat serum/donkey serum, depending on the secondary antibodies, 0.5% BSA in TBS Buffer); primary antibodies SFTPC (Abcam, Cat# ab211326, RRID: AB_2927746,1:500 dilution), anti-MUC1 antibody (Invitrogen, Cat# MA5-11202, RRID: AB_11000874, 1:200 dilution), anti-DC-LAMP antibody (Novus Biologicals, Cat# DDX0191P-100, RRID:AB_2827532, 1:100 dilution), and anti-Ki67 antibody (Abcam, Cat# ab16667, RRID: AB_302459, 1:100 dilution); secondary antibodies AlexaFluor 647 conjugated donkey anti-rabbit IgG (H+L) secondary antibody (Thermo Fisher Scientific, Invitrogen, Cat# A-31573, RRID: AB_2536183, 1:400 dilution), AlexaFluor 488 conjugated Goat Anti-Armenian hamster IgG (H+L) secondary antibody (Abcam, Cat# ab173003, RRID:AB_2936402, 1:400 dilution), and AlexaFluor 594 conjugated Goat Anti-Rat IgG (H+L) secondary antibody (Thermo Fisher Scientific, Invitrogen, Cat# A-11007, RRID:AB_10561522, 1:400 dilution). For mouse lung tissues, after IF staining and scanning, the coverslips were removed and the sections were washed twice with PBS-T. We then performed H&E staining and scanning on the same sections following the protocol described above. For quantification, based on H&E staining on same-section, 3-6 independent metastases were randomly selected and annotated for each of the analyzed tissue slices. The metastasis-vicinity regions were then defined and annotated as regions 100-200 μm away from the interfaces of these metastases. Additionally, 3-6 non-cancerous distal lung regions were randomly selected and annotated, subject to the constraint that no visible metastases were present within 250 μm. In these identified regions, cells were detected using DAPI with QuPath positive cell detection algorithm, and the percentage of MUC1-, LAMP3- and SFTPC-positive cells was then calculated, and Ki67-positive cells among total SFTPC-positive cells was calculated.

### Lung interstitial fluid extraction

Lung interstitial fluid collection was performed as previously described (12). Mice were euthanized with 50 µL of a 60 mg/mL Dolethal (pentobarbital sodium) solution (Vetoquinol) and whole lungs were collected by surgical resection, washed with blood bank saline and dried from liquid excess. Tissues were then placed in a home-made filtered centrifugation tube supplemented with a 20 µm nylon mesh filter (Repligen). 1 to 10 µL of lung interstitial fluid was collected following centrifugation at 400x g at 4 °C for 10 minutes and immediately stored on dry ice. The obtained interstitial fluid volume was used to normalize the concentration of the metabolites measured by mass spectrometry.

### Protein extraction and western blot analysis

AT2 cells were collected and washed with PBS, and lysed in RIPA buffer (Thermo Fisher Scientific, 89901) supplemented with protease (Merck Sigma, 5892970001) and phosphatase (Merck Sigma, 4906845001) inhibitors. Protein lysates were quantified with the Pierce BCA Protein Assay Kit (Thermo Fisher Scientific, 23225). Equal amounts of protein (typically 20–50 µg of protein per lane) were separated by electrophoresis on a NuPAGE 4-12% Bis-Tris gel (Thermo Fisher Scientific, NP0335BOX) in NuPAGE™ MOPS SDS Running Buffer (×20, Invitrogen, NP000102). Proteins were subsequently transferred to a nitrocellulose membrane using the iBlot2 dry transferring system (Thermo Fisher Scientific, IB301031). Transferred membranes were blocked for 1 hour at room temperature in a blocking solution of 5% milk in Tris Buffer Saline 0.05% Tween-20 (TBS-T). Subsequently, membranes were incubated overnight at 4 °C with primary antibodies: SREBP-1 (Active Motif, Cat# 39939, RRID: AB_2616606, 1:1000 dilution) and β-actin (Sigma-Aldrich Cat# A5441, RRID:AB_476744, 1:10,000 dilution). The day after, membranes were incubated with HRP-linked anti-rabbit IgG (Cell Signaling Technology, Cat# 7074S, RRID: AB_2099233) or anti-mouse IgG (Cell Signaling Technology, Cat# 7076S, RRID: AB_330924) secondary antibodies used at 1:5,000 dilution in 5% milk in TBS-T, and visualized using Pierce ECL reagent (Thermo Scientific). Images were acquired using an ImageQuant LAS 4000 (GE Healthcare). All western blot experiments were independently repeated three times.

### Mass spectrometry analysis

Metabolites extraction procedures were applied as described previously (58). Metabolite abundances were analyzed by gas chromatography (GC) tandem mass spectrometry (MS/MS). For fatty acid measurements of lung interstitial fluid samples from FSG67 treated mice, the lipid fraction (chloroform phase) was esterified as described previously (58) and separated with GC (8860 or 7890A GC system, Agilent Technologies) combined with MS (5977B or 5975C Inert MS system, Agilent Technologies). The inlet temperature was set at 270 °C and 1 μL of sample was injected into a DB-FASTFAME column (30 m × 0.250 mm) with a split ratio of 1 or 1:3 or 1:9. Helium was used as a carrier gas with a flow rate of 1 mL/min. The initial gradient temperature was set at 50 °C for 1 min and increased at the ramping rate of 12 °C min^−1^ to 180 °C, followed by a ramping rate of 1 °C min^−1^ to reach 200 °C for 1 min. The final gradient temperature was set at 230 °C with a ramping rate of 5 °C/min for 2 min and the temperatures of the quadrupole and the source at 150 °C and 230 °C, respectively. The MS system was operated under electron impact ionization at 70 eV (100–600 amu mass range). Metabolite abundances were calculated from the raw chromatograms. For relative abundance, total ion counts were normalized to an internal standard and the volume of extracted interstitial fluid. The lipids of interstitial fluid sample from *Sftpc-CreER*^*T2*^; FASN^flox/flox^ mice were measured as previously described (59), using an Agilent 7890B-7000C GC-QQQ in EI mode after derivatization of twice methanol-washed dried extracts by addition of 25 μl chloroform/methanol (2:1, v/v) and 7 μl tetramethylammonium hydroxide (TMAH (Sigma-Aldrich), room temperature, no incubation). GC-MS parameters were as follows: carrier gas, helium; flow rate, 0.9 mL/min; column, DB-5MS (Agilent); inlet, 250°C; temperature gradient, 70°C (1 min), ramp to 230°C (15°C/min, 2 min hold), ramp to 325°C (25°C/min, 3 min hold). Scan range was m/z 50-565. Data was acquired using MassHunter software (version B.07.02.1938). Data analysis was performed using MANIC software, an in house-developed adaptation of the GAVIN package (60). Metabolites were identified and quantified by comparison to authentic standards and further normalized by the volume of each interstitial fluid sample.

### Mass spectrometry imaging (MSI)

#### MALDI–MSI

4T1 cells were injected m.f.p. or i.v. into healthy 6–8-week-old female BALB/c mice. After 23 (for the m.f.p. injection) or 13 (for the i.v. injection) days, lung tissue was embedded in 3% carboxymethylcellulose (CMC), snap frozen in a cryomold dipped in liquid-nitrogen-cooled isopentane (Sigma-Aldrich). Patients’ metastatic lung samples were from the postmortem tissue donation program UPTIDER (NCT04531696, KU/UZ Leuven), and were embedded in 3% CMC. Tissues were cryo-sectioned (10 μm) using a Microm HM525 NX cryostat (Thermo Scientific), thaw-mounted onto conductive IntelliSlides (Bruker) and dried in a vacuum desiccator at room temperature for 30 min. For matrix coating, 5 mg/mL DHAP in 2:1 chloroform and methanol (v/v) was applied onto slides using an HTX M5-SprayerTM (HTX technologies) at a flow rate of 0.125 mL/min, spray nozzle temperature of 30 °C and spray nozzle velocity of 1,350 mm/min, 10 passes. MALDI–MSI was performed on a timsTOF fleX MALDI-2 (Bruker) in positive mode (m/z range 300–1800) and negative mode (250-2600), using a 50 × 50 μm or 20 × 20 μm raster size with 200 or 300 laser shots per pixel at a laser frequency of 10 kHz. Images were acquired using FlexImaging 7.0 software (Bruker). For m/z of interest, on-tissue MS/MS fragmentation spectra were acquired using a precursor ion isolation width of 1 Da and collision energy of 35 eV. Data were analyzed using SCiLS Lab 2025 (Bruker) with a mass accuracy of ±10 ppm, without denoising and applying total ion count normalization. Spectral data and regions of interest were exported to MetaboScape 2025 (Bruker). The bucket table was annotated with the Metaboscape with a build-in lipid custom list and a list of lipids obtained from MS-DIAL’s LipidBlast database was used for this purpose. The annotations for m/z of interest were manually verified against the LipidMaps database based on mass accuracy (<10 ppm) and characteristic ions observed in the fragmentation spectra (Bruker Compass DataAnalysis v.6.0). After MSI analysis, the matrix was removed by submerging the slide in 100% methanol for 30 s and the tissue was stained with H&E or IHC. Based on same-section/consecutive-section H&E staining, 3-8 independent metastases were randomly selected and annotated for each of the analyzed tissue slices. The metastasis-vicinity regions were then defined and annotated as regions 100-200 μm away from the interfaces of these metastases. Additionally, 3-6 non-cancerous distal lung regions were randomly selected and annotated, subject to the constraint that no visible metastases were present within 250 μm of these non-cancerous distal regions.

For downstream analysis of MALDI-MSI lipidomics data, spatially-resolved total ion count (TIC)-normalized abundance data for all lipid species of interest were first exported as CSV files within SCiLS Lab 2025, and further analyzed within the *R* framework (www.r-project.org). TIC-normalized data for each lipid in each given tissue section were first scaled by the maximum TIC-normalized levels for that lipid across the entire tissue section. These scaled TIC-normalized data were then further aggregated within each of the previously defined regions of interest (non-cancerous, metastasis vicinity, and metastasis), and annotated accordingly to these different region types. Aggregation was performed simply by averaging the scaled TIC-normalized data across all pixels contained in each of these regions of interest. Statistical analysis was then performed based on a linear model applied on a per-lipid basis, using *R*’s function *lm()*, with each lipid species’ aggregated and scaled TIC-normalized levels representing the response variable, and with the region type (non-cancerous, metastasis vicinity, and metastasis) treated as a categorical predictor. Effect sizes (indicating the direction and magnitude of change) and p-values (indicating significance levels for these changes) were then extracted for each lipid for the *metastasis vicinity vs non-cancerous* contrast. In cases where multiple tissue sections were analyzed concurrently, the linear model was adjusted to control for baseline differences between tissue sections, by treating the tissue section as a blocking factor. To account for multiple comparisons, p-values were in all cases subject to false discovery rate (FDR) adjustment based on the Benjamini-Hochberg method, considering all lipid species included in each analysis. A bulk lipidomics analysis of picked lung metastases and lung tissues distant to metastases was performed to confirm the distribution of key surfactant lipids from MALDI-MSI measurement in metastatic lungs from 4T1 i.v. mouse model. Due to technical limitations and the limited availability of materials from the vicinity of metastases, only lung metastases and lung tissue distant from metastases were selected for this bulk lipidomics analysis. For the representative MSI images shown in Figure 2 and Supplementary Figures 1 and 2, the white background was removed and replaced with a transparent background using the background removal tool in Microsoft PowerPoint.

#### DESI-MSI

LM1 cells were injected m.f.p. into healthy 6-8-week-old female FVB/NJ mice. Healthy mouse lungs, metastasis-infiltrated lungs and primary mammary gland tumors were snap-frozen in liquid nitrogen and stored at -80°C prior to sample preparation. Tissues were sectioned using a Leica CM3050s cryostat set to -22°C. The tissue thickness was 10 μm and tissue slices were thaw-mounted onto SuperFrost slides (ThermoFisher). Slides with thaw-mounted tissues were dried under a light stream of nitrogen gas while inside the cryostat, then placed into a polypropylene slide mailer (Leica 3802800), vacuum-sealed and stored at -80°C. Prior to MSI analysis, slides were thawed at room temperature before breaking the vacuum seal to prevent water accumulation on the tissue surface.

Desorption electro-flow focusing ionization (DEFFI)-MSI was performed using a DESI imaging source (Prosolia), which includes a 2D moving sample holder stage and a XEVO G2-XS QToF (WatersCorporation). A modified DEFFI sprayer was used (61), as well as a custom-built heated (500ºC) inlet capillary to accelerate dissolution of secondary droplets. Images were acquired using MassLynx (v.4.1, WatersCorporation) and HDImaging (v1.4) softwares. The following parameters were set for imaging: ion block temperature of 150ºC, nitrogen gas pressure of 5 bar, sprayer voltage of 4.5 kV, sprayer angle of 75º, collection angle of 10º, sprayer-to-inlet capillary distance of 3 mm and sprayer-to-surface distance of 0.5 mm. The solvent composition was 95% methanol in HPLC-grade water, and the flow rate was set to 1.5 μL per minute. The mass range for ion collection was set to 50 – 1000 m/z, the scan rate was 1 scan per second and the acquisition speed was set to 100 μm per second. The mass resolving power was 10,000 at 200 m/z, with the pixel diameter set to 100 μm.

DEFFI-MSI data was analysed using the MATLAB-based SpectralAnalysis software (62), as well as MSI data visualization script developed by Dr. Avinash Ghanate (https://avi-crick.shinyapps.io/MSIso/). Ion annotation was done using CEUMass Mediator (63). WGCNA analysis was performed to determine tissue areas of similar chemical composition (modules), as described previously (bioRxiv 2018.01.09.230052) (64). A “lung-specific” module was defined, which consisted of ions enriched in lung tissue (both metastatic and healthy) compared to mammary gland tumor and their metastases within lung tissue. The subsequent comparison between healthy and metastatic lungs was constrained to the “lung-specific” ions only.

#### OrbiSIMS

Metastasis-infiltrated lung samples from the LM1 model, as well as wildtype lungs from age-matched FVB/NJ female mice were prepared as described for DESI-MSI above, with the exception of thaw-mounting the tissues onto indium tin oxide (ITO)-coated slides (Bruker). Samples were imaged using an OrbiSIMS (ION-TOF GmbH, Münster, Germany) equipped with a time-of-flight (ToF) mass analyzer and an Orbitrap Q Exactive HF mass analyzer (Thermo Fisher, Bremen, Germany) (15). All acquisitions were performed using the Orbitrap and a 20 keV Ar_3200_^+^ GCIB as a primary ion source. Mass calibration of the Orbitrap was performed on the day of analysis using silver cluster secondary ions. Negative polarity secondary ion images were acquired using the GCIB with a current of 15.0 pA selected using a beam duty cycle of 18.18% with a 200 µs cycle time. The Orbitrap mass range was m/z 80−1200 and was operated with a mass-resolving power of 240,000 using a transient time of 500 ms. The automatic gain control is switched off and an injection time set to match the transient time. The collisional cell was set-in low-pressure mode with a pressure of 4.1E-02 mbar. The target potential, VT, was set at -32.3 V according to the procedure in Matjacic et al (65). The field of view was 500 μm × 500 μm (175 × 175 pixel). For all acquisitions, an electron floodgun with a current of -20 µA was used to compensate charging over the surface of the sample complemented by Ar gas flooding at a pressure of 7.7×10^-7^ mbar. Ion images were generated by selecting the ion of interest from the mass spectrum. For each image, a 4-pixel binning was applied. The Images were normalized to the total ion count of the image (also 4 pixels binned). IONTOF SurfaceLab 7.3 was used to acquire the data and IONTOF SurfaceLab 7.4 was used to process the results and assign the peaks.

### Lipidomics analysis

The 6-week-old female BALB/c mice were inoculated with 4T1 cells (i.v. 1 × 10^6^ cells). After 12 days, lung metastases and lung tissues distant to metastasis were collected, snap frozen under liquid-nitrogen conditions. Lipids were extracted by mixing 700 μl in water, homogenizing (Precellys, Bertin) with 800 μl 1 N HCl:CH3OH 1:8 (v/v), 900 μl CHCl3, 200 mg/mL of the antioxidant 2,6-di-tert-butyl 4-methylphenol (Sigma-Aldrich) and SPLASH LIPIDOMIX Mass Spec Standard (330707, Avanti Polar Lipids). After vortexing and centrifugation, the lower organic fraction was collected and evaporated in a speedvac. Lipid species were analyzed by LC–ESI/MS/MS as previously described (12) on a Nexera X2 UHPLC system (Shimadzu) coupled with hybrid triple quadrupole/linear ion trap MS (6500+ QTRAP system; AB SCIEX). Peak integration was performed with the MultiQuant software (v.3.0.3). Lipid species signals were corrected for isotopic contributions calculated using Python Molmass v.2019.1.1 and quantified based on internal standard signals and adhering to the guidelines of the Lipidomics Standards Initiative (level 2 type quantification).

### Formate concentration measurement

The formate detection method was adapted from a published protocol for short chain fatty acids quantification (66). Samples were derivatized in five replicates of metastasis-conditioned media from two independent batches, plastic blank controls (LC-MS grade H_2_O treated as sample) and media-only controls. Additionally, two six-point calibration curves were prepared for absolute quantification of formate in media samples and plastic blank samples, respectively. In brief, samples were thawed on ice, and 40 μL was placed in an Eppendorf tube on ice. Then, the samples were spiked with 0.5 nmol D^3^-Acetate as internal standard. After vortexing vigorously, the samples were submitted to derivatization. For the six-point calibration curve, 40 μL from 7 random media samples from the study set was mixed together to constitute media stock. Various volumes of a labelled ^13^C, D-formate solution and medium stock were dispensed into Eppendorf tubes for a final volume of 40 μl in order to get formate concentrations ranging from 25 μM to 500 μM. A similar strategy was applied to build a calibration curve in plastic blank stock. Both samples and calibrants were submitted to derivatization as follows: 100 μL of ice-cold derivatization mix (60 mM 1-ethyl-3-(3-dimethylaminoproryl)carbodiimide (EDC) in methanol, 4% Pyridine in methanol, 125 mM 3-nitrophenylhydrazine (3-NPH) in methanol, 1:1:3 (v/v/v)) were added to the medium. After vortexing, the samples were incubated at 4 °C for 60 min. Then, the samples were spin down at 12.000 g for 10 min at 4 °C. Twenty microliters of supernatant were transferred into a LC-MS vials equipped with an insert containing 200 µl of a 500 µM β-mercaptoethanol solution for quenching. The samples were subsequently spin down at 12.000 g for 10 min at 4 °C, and transferred to the LC-MS for data acquisition on the same day. Formate analysis was performed by injecting 5 µL samples into a Vanquish® Horizon UHPLC system (Thermo Scientific) coupled to an Exploris® 480 Orbitrap® mass spectrometer (Thermo Scientific). Chromatography was carried out on a Waters Acquity BEH C18 column (2.1×100 mm, 1.7 µm) equipped with a Waters Acquity BEH pre-column. The temperature of the column was kept at 60 °C. The mobile phases consisted of water (Eluent A) and methanol (Eluent B). Loading and gradient occurred at 0.2 mL/min flow rate; loading step last 1 min with 10% B; then started a linear gradient ranging from 15% B to 30% B in 2 min, then from 30% B to 100% B in 1 min; at 5 min started the regeneration step with 100% B; from 7 min until 7.2 min, mobile phase composition was brought back to 10%. The gradient was stopped at 8.50 min. The MS experiment on the Exploris® 480 Orbitrap® was performed using heated electrospray ionization (HESI) with polarity switching enabled. Source parameters were applied as follows: sheath gas flow rate, 55; aux gas flow rate, 10; sweep gas flow rate, 0; static spray voltage, 3.5 kV (+) / 3.2 kV (-); Ion transfer tube temperature, 325 °C; Vaporizer temperature, 100 °C. The Orbitrap mass analyzer was operated at a resolving power of 480,000 (at 200 m/z) in full-scan mode (scan range: m/z 70-1000; S-lense RF level, 50%; normalized automatic gain control (AGC) target set to 70% with a maximum injection time: 100 ms). Full scan data were acquired in centroid and negative mode. The data were acquired with the Thermo Xcalibur software (Version 4.7.69.37) and analyzed using TraceFinder (Version 5.1).

### UPTIDER patient program

UPTIDER is a monocentric post-mortem tissue donation program for patients with end-stage breast cancer (NCT04531696, local ethics number: S64410, approval 30th November 2020 by ethical committee research UZ/KU Leuven). Written informed consent is obtained from all participants and all relevant ethical regulations including the Declaration of Helsinki are complied with. Patients with metastatic breast cancer who consent to participate undergo a rapid research autopsy in the first 12 h after death. Tissue samples were embedded in 3% carboxymethylcellulose (CMC), snap frozen in a cryomold dipped in liquid-nitrogen-cooled isopentane and processed for MALDI-MSI measurement, as described above. IHC was performed from paraffin embedded patient tissues, as described above. Clinicopathological information for every patient in the UPTIDER dataset is shown in Supplementary Figure 1a.

### Spatial transcriptomics using visium spatial gene expression (10x genomics)

#### 4T1 m.f.p. metastatic lung sample preparation for spatial transcriptomics analysis

Visium spatial transcriptomics analysis was carried out according to the manufacturer’s instructions (10x Genomics) with the following parameters: Fresh-frozen mouse metastatic lung tissue derived from 4T1 m.f.p. injection model was processed for spatial gene expression profiling using the 10x Genomics Visium Spatial Gene Expression platform (Visium Slide PN-2000233, SN: V13J17-305). Tissue sections were mounted on a Visium Spatial Gene Expression Slide with four capture areas, each containing ~5,000 spatially barcoded spots. Following fixation and hematoxylin and eosin (H&E) staining, tissue permeabilization was performed for 18 minutes—a duration determined through prior tissue optimization using the 10x Genomics Tissue Optimization protocol (CG000238). mRNA released from permeabilized tissue was hybridized to oligonucleotides on the slide, and reverse transcription was carried out in situ to generate spatially barcoded cDNA. Subsequent library preparation followed the manufacturer’s protocol (Visium Spatial Gene Expression Reagent Kits User Guide, CG000239 Rev F), including second-strand synthesis, cDNA amplification, enzymatic fragmentation, end repair, adaptor ligation, and sample index PCR. Libraries were sequenced on an Illumina platform (NovaSeq 6000) using paired-end sequencing (Read 1: 28 bp, Read 2: 90 bp) at a target depth of (50k reads per cell/spot).

#### Data processing

We used the PhiSpace R package (21), a novel reference-based cell type annotation method for single-cell and spatial omics data, to deconvolute cell types present in each Visium spot. We used the previously published scRNA-seq data from 4T1 cells derived metastatic lung as reference (22). Both scRNA-seq and Visium data were log1p normalized using PhiSpace::logTransf. Cell type deconvolution was then conducted using the partial least squares regression model PhiSpace::PhiSpaceR_1ref(phenotypes = “CellType”, PhiSpaceAssay = “log1p”, regMethod = “PLS”). After cell type deconvolution, each Visium spot was scored against all cell types defined in the scRNA-seq reference. To incorporate spatial information into these scores, we then conducted spatial smoothing of these scores using PhiSpace::spatialSmoother(smoothReducedDim = T, x_coord = “x”, y_coord = “y”). We then applied k-means clustering based on these spatially-aware cell type scores to identify 5 spatial niches. Among these spatial niches, one clearly corresponded to metastatic cores (70 Visium spots in total), as validated by both cell type annotation and H&E image (Supplementary Figure 3d), whereas another matched the vicinities of metastases (111 Visium spots in total), with the remaining 3 spatial niches corresponding to metastasis-distal regions (919 Visium spots in total).

To understand how the metabolism of AT2 cells differ between the metastasis-vicinity and metastasis-distal regions, we ranked all 1100 Visium spots based on their AT2 PhiSpace scores, and annotated those spots with scores in the top 20% percentile as ‘AT2-enriched’ spots, resulting in 33/187 ‘AT2-enriched’ spots in the metastasis-vicinity/distal regions, respectively. Of note, no ‘AT2-enriched’ spots were found in the metastatic-core region based on this criterion.

#### Gene set enrichment analysis (GSEA) using Visium data

Differential expression analysis, based on comparing Visium-derived gene expression levels between ‘AT2-enriched’ spots in the metastasis-vicinity and metastasis-distal regions, was performed within the *Seurat* (v4.1.0) (www.satijalab.org/seurat) framework (67), using the function *FindMarkers* with default parameters (other than preserving all genes in the output regardless of their determined fold-changes and/or their fraction of expressing spots across the two groups of interest). The resulting (vicinity ‘AT2-enriched’ *vs* distal ‘AT2-enriched’) log fold changes (log_2_ FC) and their associated p-values for each gene were then combined with the maximum fraction of spots with non-zero expression for that gene among the two groups being compared (Xmax, where Xmax = 1 for genes expressed in 100% of spots in either of the two groups), to derive an adjusted ranking metric RM_adj_ = ™ log_2_ FC × log_10_(p-value) × X_max_, whereby highly positive/negative values of RM_adj_ indicate respectively genes with highly up/downregulated expression in ‘AT2-enriched’ spots in the metastasis-vicinity region (relative to those in the metastasis-distal region). Pre-ranked gene-set enrichment analysis (GSEA; https://www.gsea-msigdb.org/gsea) (68) was then performed, using the *R* package *fgsea* (bioRxiv 2021.02.01.060012) (v1.20.0; https://github.com/ctlab/fgsea; multilevel implementation with 10000 initial permutations and no lower bound for p-value estimation) based on this ranking metric RM_adj_, and considering a collection of 3066 mouse gene sets. The latter comprised all HALLMARK and CURATED (C2) gene sets in the Broad Institute Molecular Signatures Database (MSigDB; https://www.gsea-msigdb.org/gsea/msigdb) (69,70) except for those in category C2:CGP, all of them obtained via the *R* package *msigdbr* (v7.4.1; https://igordot.github.io/msigdbr), plus all KEGG (https://www.genome.jp/kegg) (71) metabolism gene sets, obtained by querying KEGG’s REST API (https://www.kegg.jp/kegg/rest). Only gene sets containing between 3 and 1500 genes were considered in this analysis. For the purpose of assessing the proportion of up- and down-regulated gene sets related to proliferation/cell cycle, migration, and IL-6 signaling, the GSEA results were filtered based on the following regular expressions: “PROLIF|CELL(_*)CYCLE|_(G1|G2|G2M|M|S)_(PHASE|CHECKPOINT)|MITOSIS|M ITOTIC|(ANA|META|PRO)PHASE” (for proliferation and/or cell cycle-related gene sets), “MIGRAT|CHEMOTAX|CHEMOKIN|CHEMOATTRACT” (for migration-related gene sets), and “(_INTERLEUKIN|_IL)_*6” (for IL-6 signaling-related gene sets). The results for these gene-set subsets were then classified into 4 categories based on their normalized enrichment score (NES) values: either (a) strongly (NES >1) or (b) mildly (0.5 < NES < 1) up-regulated in ‘AT2-enriched’ spots in the metastasis-vicinity region (relative to their metastasis-distal counterparts), or (c) mildly (-1 < NES < -0.5) or (d) strongly down-regulated (NES < -1) in ‘AT2-enriched’ spots in the metastasis-vicinity region (again, relative to their metastasis-distal counterparts).

### Single-cell RNA-seq, data pre-processing and cell-type assignment

#### LM1 m.f.p. metastatic model sample preparation for scRNA-seq

LM1 model-derived primary mammary gland tumors and metastatic lungs and healthy mouse lungs were collected and minced using blades. Tissue dissociation was performed in 2 mL RPMI media containing 0.3 mg/mL liberase (Roche) and 1 µg/mL DNase1 at 37 °C for 45 minutes with occasional vortexing. The dissociated lungs were quenched by adding 10 mL of washing buffer (PBS supplemented with 3% FBS and 2 mM EDTA). After gentle mixing, the resuspended cells were passed through a 70 µm cell strainer and centrifuged at 300x g for 5 minutes. Following an additional washing step, each cell pellet was resuspended in 2 mL of red blood cell lysis buffer (Merck) and incubated in a 37 °C water bath for 3 minutes. After adding 20 mL of washing buffer, the suspension was passed through a 40 µm cell strainer and centrifuged again at 300 × g for 5 minutes. The resulting cell pellets from tumors and lungs were resuspended in PBS with 1% BSA. A 150 µL (1000 cells/µL) was transferred to LoBind Tubes (Eppendorf, 022431021) for viability QC testing using the LUNA-FX7. Once the isolated cells passed viability QC test (>80%), the samples were processed through making emulsion and library preparation steps. The sequencing library was prepared from 10,000 cells. Each tumor sample was analyzed separately. Cells from two lungs were pooled together for each lung sample.

#### Data processing

The single-cell RNA-sequencing (scRNA-seq) data based on the experimental (only lung metastases without primary tumor; i.v. injected) metastasis model was previously analyzed in (22), and the corresponding data and cell-type annotations are available in the Gene Expression Omnibus (GEO), under accession number GSE236084. For the purpose of the present study, only data corresponding to mice not treated with tumor-secreted factors, either before cancer-cell injection (healthy lungs, CM0) or 16 days post cancer-cell injection (metastatic lungs, CM2) were used.

For LM1 m.f.p. metastatic model, sample-level count data were generated using from the fastq files using 10x Genomics Cell Ranger 7.1.0 against 10X mouse reference transcriptome refdata-gex-mm10-2020-A. Reads were counted against both the intronic and exonic parts of the transcriptome. The resulting filtered feature-barcode matrices were used as input into R for further analysis using the Seurat package with default settings unless otherwise stated (67). Count matrices were then normalised per cell using a centered log ratio (CLR) transformation [Seurat::NormalizeData(normalization.method = “CLR”, margin = 2)]. The top 2000 most variable genes that exhibited high cell-to-cell variation in the dataset were identified [Seurat::FindVariableFeatures(selection.method = “vst”, nfeatures = 2000)]. Data were scaled and centred using all available features [Seurat::ScaleData] such that the mean expression across cells was 0 and the variance was 1 in order to ensure that highly-expressed genes did not dominate downstream analysis. Principal component analysis (PCA) dimensionality reduction was run using the 2000 most variable genes to assess the dimensionality of the dataset. The non-linear dimensional reduction technique UMAP was then applied using the first 30 PC so that similar cells were placed together in low-dimensional space. A k-nearest neighbours (KNN) graph was constructed from the Euclidean distance in PCA space, refining edge-weights between cells based on shared overlap of local neighborhoods (Jaccard similarity) [Seurat::FindNeighbors(dims = 50, k.param = 20, compute.SNN = TRUE)]. The KNN-graph was used to group cells into clusters showing similar expression profiles via the Louvain algorithm [Seurat::FindClusters(resolution=0.5)]. The effects of cell cycle heterogeneity were assessed by scoring each cell based on the expression of the default canonical G2/M and S phase marker genes from previous study loaded with Seurat [Seurat::CellCycleScoring] (72). Potential doublets cells were flagged, though not removed, using the R package DoubletFinder’s doubletFinder_v3 function (73), assuming a doublet rate of 7.5%. After each sample dataset had been separately processed, a set of integration features were selected that represented genes repeatedly variable across them all [Seurat::SelectIntegrationFeatures]. Integration anchors, i.e. cross-dataset pairs of cells matched in biological state, were identified using reciprocal PCA (rPCA) focusing on just the integration features. The anchors were used to integrate the sample datasets into a single object using the Seurat::IntegrateData function with default settings. The integrated data were then centred and scaled, before PCA and UMAP dimensionality reduction, KNN-graph construction and Louvain clustering using the same parameters as for the single samples (see above). Automated cell-type annotation was performed on the raw count data, against gene signatures from the Mouse Cell Atlas using the R package scMCA v0.2.0 (74). Preliminary unsupervised cluster cell-type identity was assigned based on the most common cell-level annotation discovered in each and further refined based on manual review of known marker genes. AT2 cells were defined by the expression of the following genes: SFTPB, SFTPC, SFTPD, MUC1, ETV5.

#### SCENIC analysis

SCENIC analysis was performed as described previously (75) using the pySCENIC (0.12.1). The required databases for running SCENIC, including the transcription factor (TF) database (cisTarget.mm10.mc_v10_clust.feather) and motif annotation database (motifs-v10nr_clust-nr.mgi-m0.001-o0.0.tbl), were downloaded from the cistarget website (https://resources.aertslab.org/cistarget/). The input matrix of pySCENIC was the raw count matrix output from Seurat. And the data was downsampled by randomly selecting 318 cells from cell clusters containing more than 318 cells in the scRNA-seq dataset derived from 4T1 (i.v.) metastatic model and 500 cells from cell clusters with more than 500 cells in the scRNA-seq dataset derived from LM1 (m.f.p.) metastatic model. The co-expression modules are inferred using GRNBoost2. The activity of the regulatory networks was evaluated across the entire dataset by calculating the Area Under the Recovery Curve (AUC) for the predicted set of target genes associated with each transcription factor (TF).

### Statistics and reproducibility

Statistical analyses were conducted using GraphPad Prism V10 (GraphPad Software) or within the R/Bioconductor framework. Data are presented as mean ± SEM as indicated in the figure legends. Details on statistical tests are indicated in the figure legends. P values are shown in each figure, with those less than 0.0001 represented as P < 0.0001. Data normality was assessed using the Shapiro–Wilk and Kolmogorov–Smirnov tests, and parametric or nonparametric tests were applied accordingly. All parametric and nonparametric tests were two-tailed, with P < 0.05 considered statistically significant. Mathematical outliers were determined using the Grubbs’ or ROUT method of regression (GraphPad) with alpha = 0.05 or coefficient Q = 1%.

### Materials Availability

This study did not generate new unique reagents, except of genetically manipulated cell lines based on commercially available constructs. Reagents generated in this study will be made available on request through the lead author or the collaboration partner that generated the resource, but we may require a payment and/or a completed Materials Transfer Agreement if there is potential for commercial application.

## Supplementary Material

1

2

3

## Data Availability

Mouse single-cell RNA-sequencing data generated and deposited in the Gene Expression Omnibus (GEO) under accession number GSE287197. Mouse Visium data has been deposited in the GEO under accession number GSE318532. The publicly available mouse single-cell RNA-sequencing dataset GSE236084 can be accessed and downloaded from the GEO https://www.ncbi.nlm.nih.gov/geo/query/acc.cgi?acc=GSE236084. All other data supporting the findings of this study are available within the Article and the Supplementary Information, and from the corresponding author on reasonable request.

## Statement of significance

Current therapies in oncology targeting the cancer or immune cell compartment of tumors show limited efficacy against breast cancer-derived metastases. We discovered that decreasing the lipid metabolism of lung resident AT2 cells is sufficient to impair lung metastasis growth in mice without apparent adverse effects.

## Code Availability

No original software and/or algorithms were developed in the present study; however, code used for data analysis can be provided upon request. Any additional information required to reanalyze the data reported in this paper is available from the corresponding author upon request.
