# Supplementary material for: Targeting the lipid metabolism proteins FASN and GPAM in alveolar type II cells decreases lung metastasis
Source: Cancer Discov. Author manuscript; Available in PMC 2026 Jun 12. (PMC7619144; doi:10.1158/2159-8290.CD-25-0191)
Supplement: 3 [file EMS212901-supplement-3.pdf]

Supplementary Figure 1

a

Clinical data of patients from the UPTIDER program

Purpose	Patient ID	Age diagnosis	Age death	Molecular subtype primary tumor	Histotype	Grade of tumor at diagnosis*	Stage at diagnosis*
IHC from FFPE tissues	2004	56	61	ER+/PR+/HER2-	Metaplastic-SCC	2	II
	2009	51	62	ER+/PR+/HER2-	NST	3	II
	2014	48	56	ER+/PR+/HER2-	NST	1 or 2	IV
	2024	36	41	TNBC	NST	3	IIA
	2026	47	91	ER+/PR+/HER2-	NST	NA	IIIA
MSI	2014	48	56	ER+/PR+/HER2-	NST	1 or 2	IV
	2004	56	61	ER+/PR+/HER2-	Metaplastic-SCC	2	II
	2030	41	65	ER+/PR+/HER2-	NST	2	II

*The stage at time of sample acquisition was stage IV for all UPTIDER patients

b

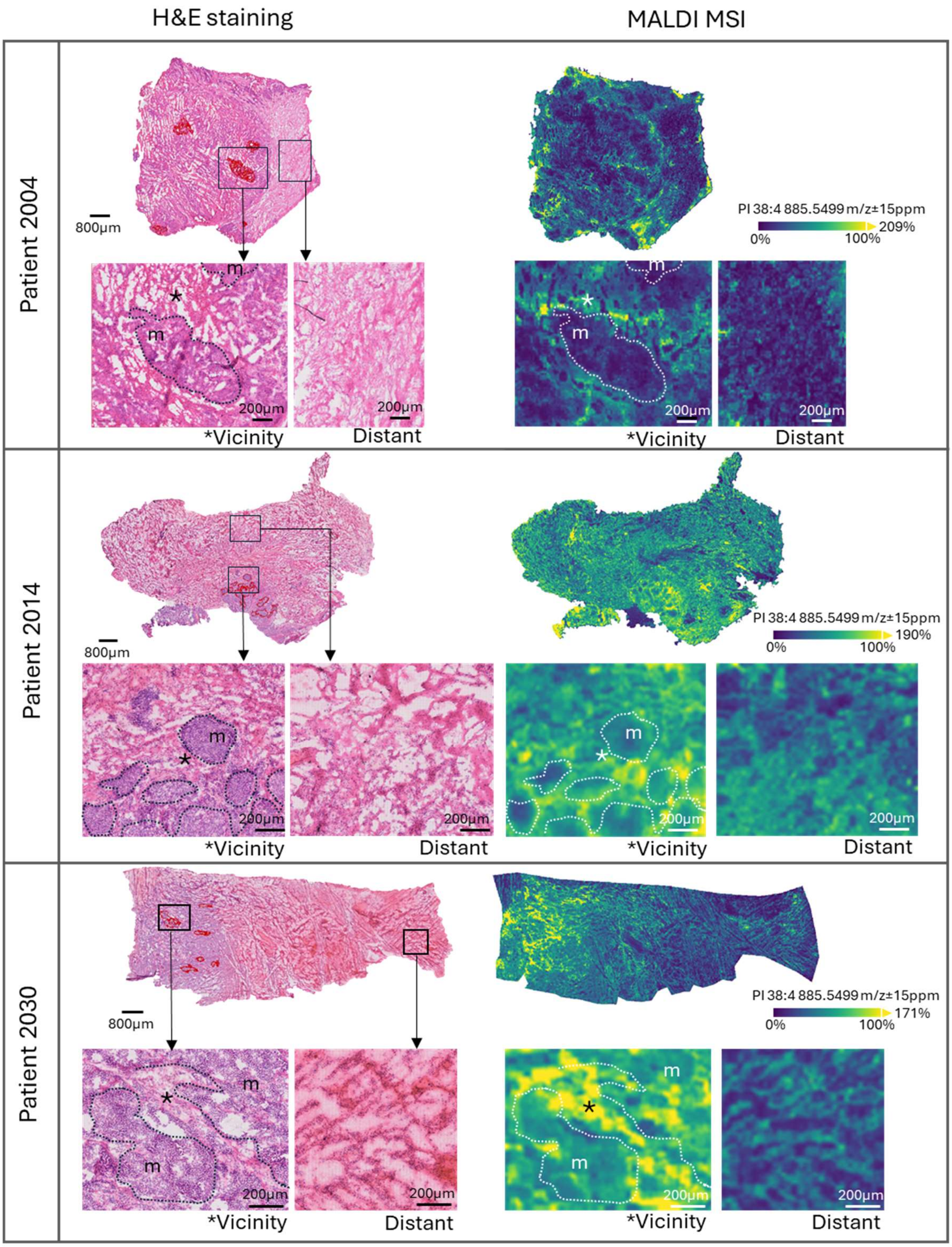

Supplementary Figure 1 Surfactant lipids are enriched in the vicinity of lung metastases from patients with breast cancer

a. Clinicopathological information of patients in the UPTIDER dataset used for immunohistochemistry staining (IHC) and MSI analyses. Abbreviations: ER, estrogen receptor; PR, progesterone receptor; TNBC, triple negative breast cancer; SCC, squamous cell carcinoma; NST, no special type.

b. Representative total ion count-normalized MALDI-MSI ion images of metastatic lung tissues from breast cancer patient 2004, 2014 and 2030 of the UPTIDER program, alongside an optical image of the H&E staining of the same tissue section. Lung metastases are outlined with red lines on the H&E staining. In the zoomed-in views, metastasis (m) are outlined with dash lines on both H&E and MSI images, and stars indicate the vicinity of metastases. Scale bars: 800 µm (overview) and 200 µm (zoomed-in view).

Supplementary Figure 2

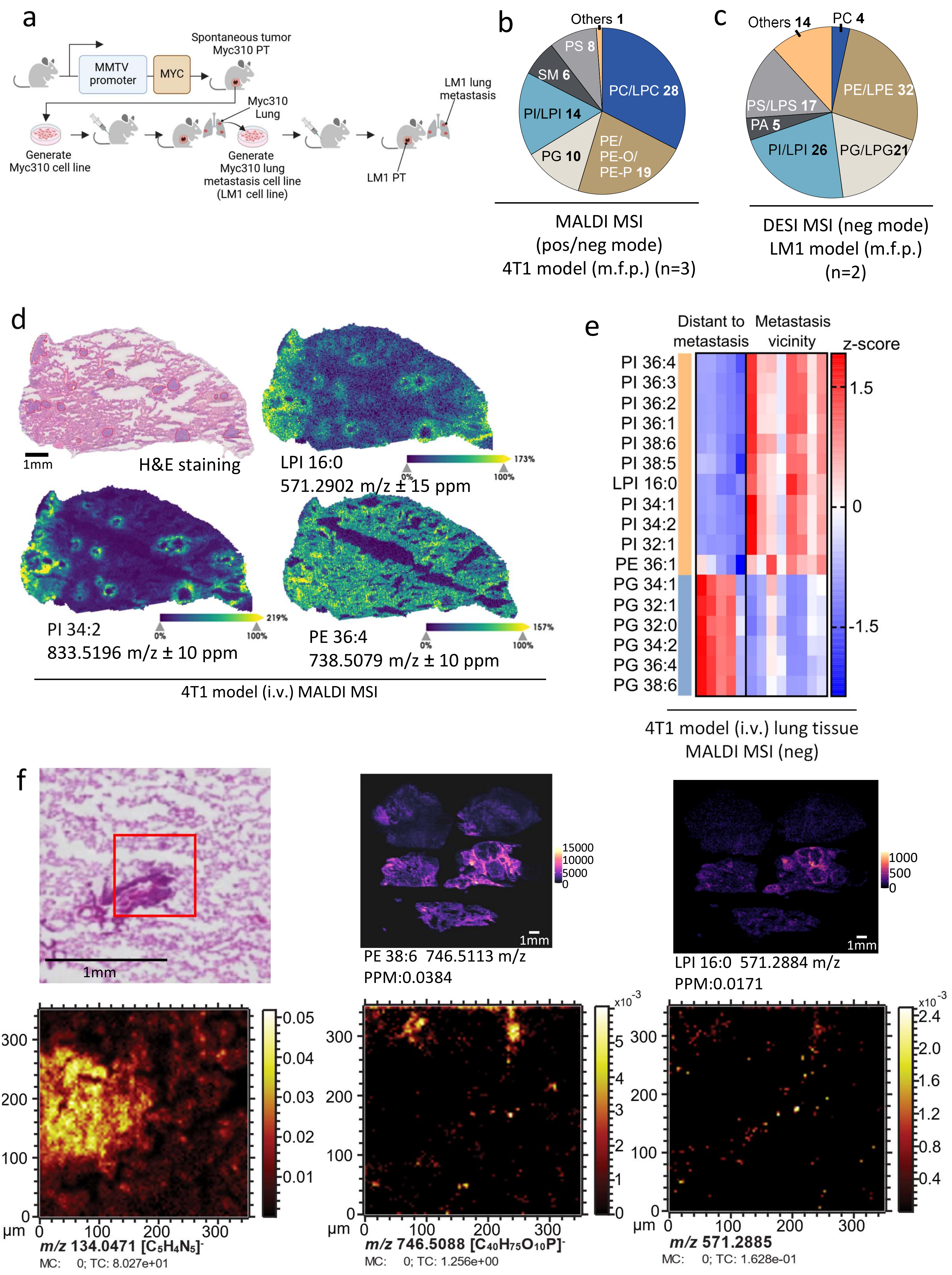

Supplementary Figure 2 AT2 cells and surfactant lipids co-localize in the vicinity of metastases from mice

a. Schematic illustration of the establishment of LM1 cell line and LM1 m.f.p. injection-derived metastatic mouse models. PT represents the primary tumor.

b. Pie charts showing the number of detected lipids in metastatic lungs from 4T1 mammary fat pad injected mouse models, as measured by MALDI-MSI. Pos and neg mode represent the positive and negative ion mode in the MALDI-MSI detection settings, respectively. 86 lipid species were identified (34 by positive mode; 52 by negative mode)

c. Pie charts showing the number of detected putatively-identified lipids in metastatic lungs from LM1 mammary fat pad injected mouse models, as measured by DESI-MSI. Neg mode represent the negative ion mode in the DESI-MSI detection settings.

d. Representative total ion count-normalized MALDI-MSI ion images of metastatic lung tissues from 4T1 i.v. injection derived lung metastasis, alongside an optical image of the H&E staining of the same tissue section as from a previous study (12). Lung metastases are outlined with red lines on the H&E staining. Scale bars represent 1mm.

e. Heatmap showing z-score distributions of region-aggregated, scaled total ion count (TIC)-normalized abundances for lipid species among the 32 identified in the overlap area of **Figure 2a** and further found to be significantly altered between lung tissues distant to metastases and metastasis-vicinity regions, as measured by negative-polarity MALDI-MSI in a metastatic lung tissue sample from one mouse with 4T1 (i.v.) derived lung metastases. Each row represents a distinct lipid species, whereas each column represents each of the individual metastasis-distant or metastasis-vicinity regions annotated for this mouse. The color code indicates z-scores based on region-aggregated, scaled total ion count (TIC)-normalized abundances for each lipid species in each of these regions (see Methods), with the z-score transformation performed on a per-lipid basis, across all shown regions. Lipid-level alteration magnitudes and their statistical significance were determined based on a linear model applied on a per-lipid basis, with TIC-normalized abundances for each lipid species first scaled relative to their maximum value within the tissue section, then aggregated within each individual region ($n = 5$ regions for lung tissue distant to metastasis, and $n = 8$ regions for metastasis vicinity), and finally modeled as a function of the region type (treated as a categorical predictor; see Methods). Only those lipid species showing FDR-adjusted p-values below 0.05 for the metastasis vicinity vs lung tissue distant to metastasis contrast are shown in the plots. Orange/blue bars indicate metabolites that are significantly higher/lower in the metastasis vicinity compared to lung tissue distant to the metastasis, respectively.

f. Representative total ion count-normalized OrbiSIMS single ion images (bottom panel) of a metastatic border (marked with a red square) in lungs from mice with m.f.p. injections of LM1 cells, alongside an optical image of the H&E staining of the same tissue section. DESI-MSI images of the same ions (upper panel) are shown for reference (LPI16:0 was already shown in **Figure 2c**). High nucleotide fragment signal (m/z 134.0471) defines a metastatic lesion.

(a. Created with BioRender.com)

Supplementary Figure 3

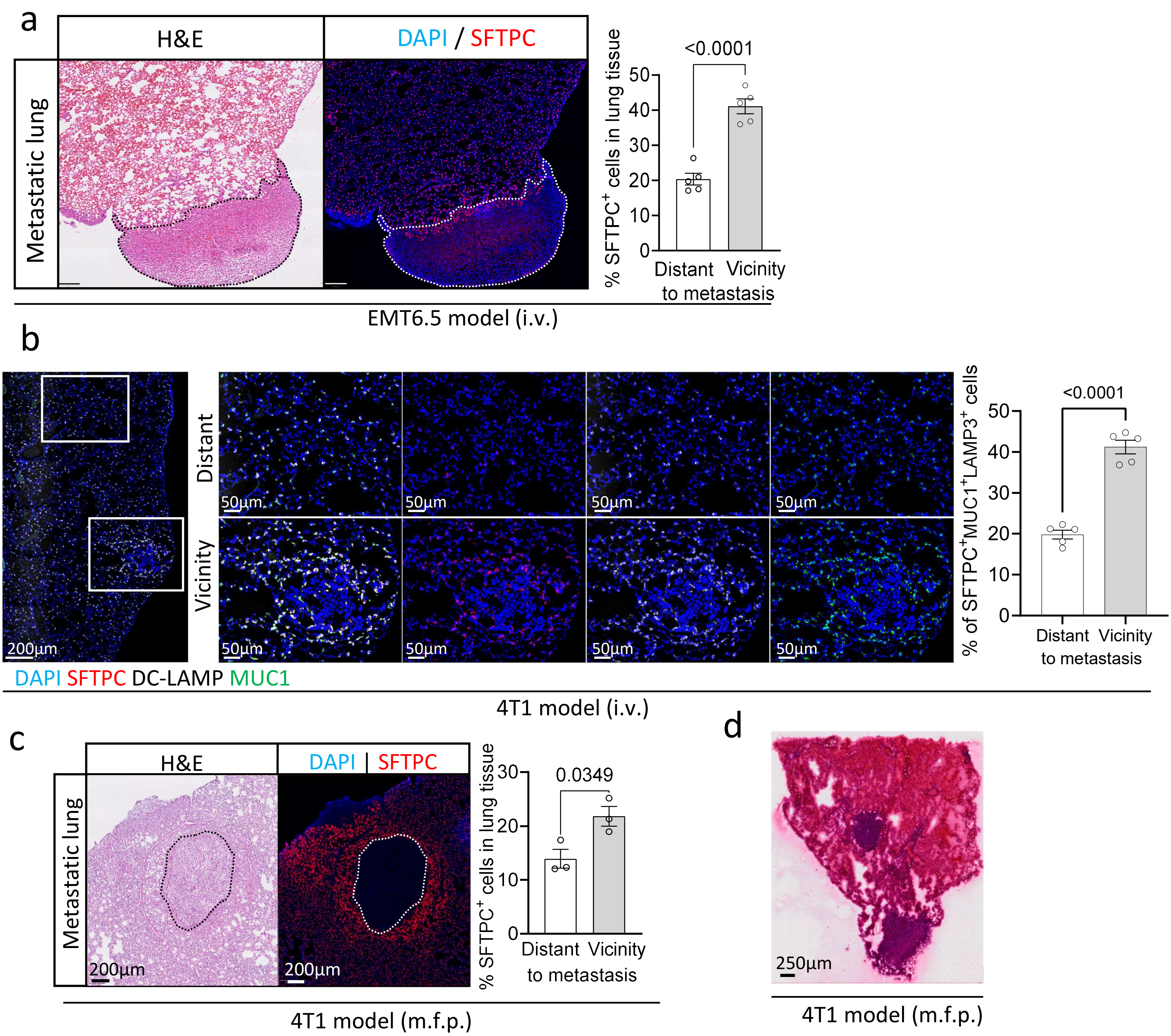

Supplementary Figure 3 Metastases progression increases the enrichment of AT2 cells

a. Representative IHC staining for SFTPC (red) and DAPI (blue) in lung metastases derived from EMT6.5 intravenous injection mouse model, alongside an optical image of the H&E staining of the same tissue section. Scale bars represent 200 µm. The percentage of $SFTPC^{+}$ cells is shown as mean $\pm$ SEM of five biological replicates on the right panel. Statistical significance was determined using unpaired two-tailed t-test.

b. Representative IF staining of AT2 cell markers SFTPC (red), LAMP3 (white), MUC1 (green) and DAPI (blue) in the metastatic lung tissues from 4T1 i.v.-injected mouse models. Zoomed-in views show regions in the vicinity of metastasis and distant lung areas. The first zoomed-in panel shows the overlay of SFTPC, MUC1 and LAMP3. Scale bars: 200 µm (overview) and 50 µm (zoomed-in views). The percentage of SFTPC, MUC1 and LAMP3 positive cells is shown as mean $\pm$ SEM of n=5 mice on the right panel. Statistical significance was determined using unpaired two-tailed t-test.

c. Representative IHC staining for SFTPC (red) and DAPI (blue) in metastatic lung tissues derived from 4T1 m.f.p. injected mouse models, alongside an optical image of the H&E staining of the same tissue section. The metastatic lesion is outlined with dash lines. Scale bars represent 200 µm. The percentage of $SFTPC^{+}$ cells is shown as mean $\pm$ SEM of n=3 mice on the right panel. Statistical significance was determined using unpaired two-tailed t-test.

d. H&E staining of lung tissue prior to performing Visium transcriptomics analysis (**Figure 4a**). Scale bar represent 250 µm.

Supplementary Figure 4

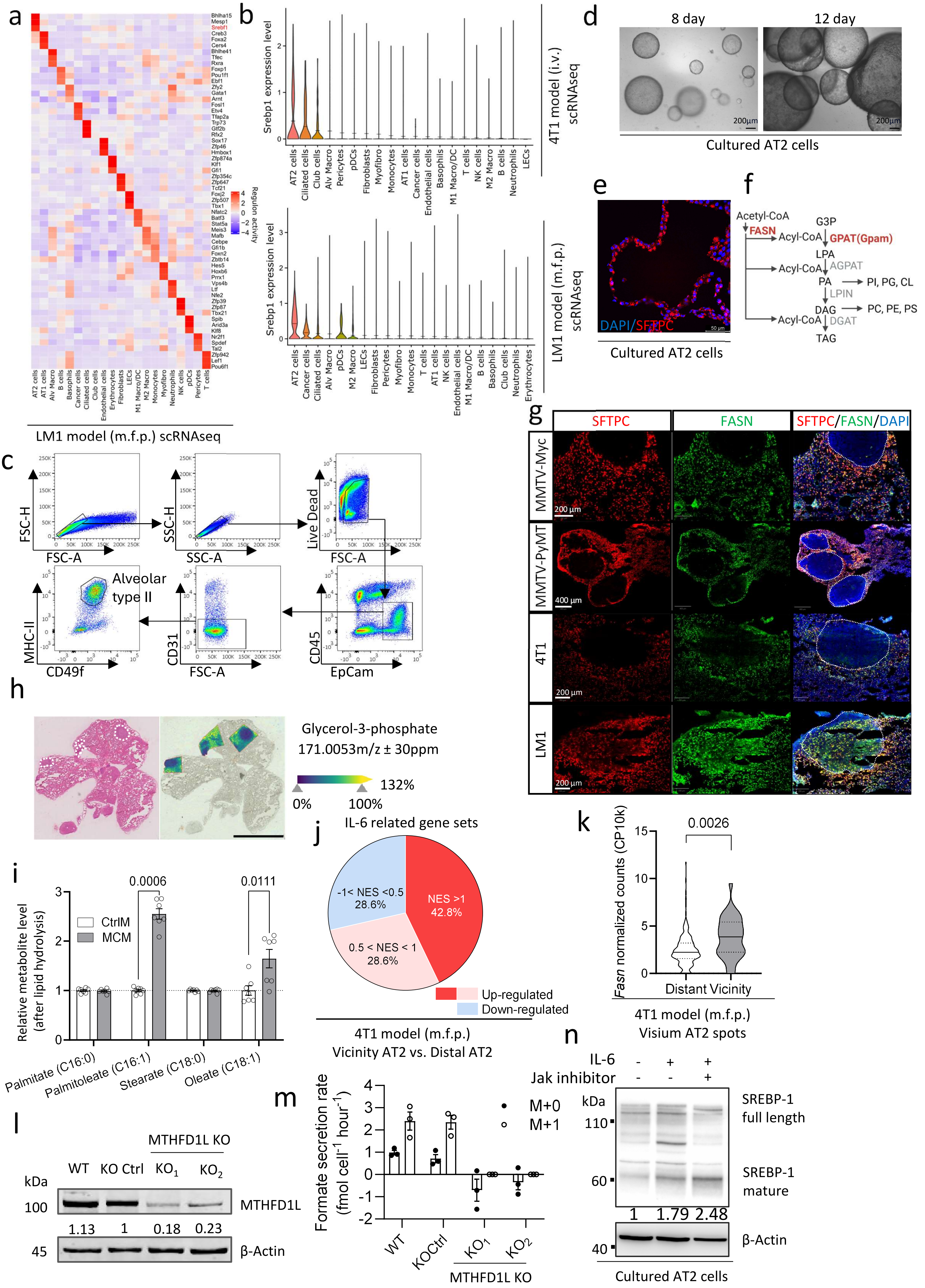

Supplementary Figure 4 The metastasis secretome reprograms AT2 cell lipid metabolism by activating SREBP-1

a. Heatmap showing the top three most active regulons compared to all other cell types. Regulon activity (AUC scores) was calculated using AUCell implemented in SCENIC on scRNA-seq data of healthy and LM1 m.f.p. injection-derived metastatic lungs. The average AUC score was calculated across all cells belonging to each cluster. The resulting matrix was z-score normalized per regulon across cell types. Each row represents a distinct regulon, and color intensity reflects its z-score normalized activity.

b. Log-normalized *Srebp-1* expression distributions across different lung cell types, based on scRNA-seq data of healthy and 4T1 i.v.-derived metastatic lungs (upper panel), and healthy and LM1 m.f.p. injection-derived metastatic lungs (bottom panel).

c. Gating strategy for isolating AT2 cells using fluorescence-activated cell sorting (FACS).

d. Representative images of the *ex vivo* cultured AT2 cells at 8 and 12 days post-seeding. Scale bars represent 200μm.

e. Representative immunofluorescence staining for SFTPC (red) and DAPI (blue) in cryosectioned *ex vivo* cultured AT2 cells. Scale bars represent 50μm.

f. Schematic representation of metabolic pathways involved in lung surfactant production.

g. Immunofluorescence staining for SFTPC (red), FASN (green) and DAPI (blue) in metastatic lungs derived from MMTV-Myc and MMTV-PyMT spontaneous breast cancer mouse models, and 4T1 and LM1 m.f.p. injection mouse models. Scale bars represent 200μm for MMTV-Myc, 4T1 and LM1 models and represent 400μm for MMTV-PyMT model. Metastatic lesions are marked by white dash lines in the merged images.

h. Representative total ion count-normalized MALDI-MSI signal of Glycerol-3-phosphate on metastatic lung tissues from 4T1 m.f.p. injection mice (three metastatic regions are measured), alongside an optical image of the H&E staining of the same tissue section. Lung metastases are outlined with white dash lines on the H&E staining. Scale bars represent 2mm.

i. Relative fatty acid abundance in the control medium (CtrlM) and metastasis conditioned medium (MCM) samples (n = 7/condition). Data shown as mean $\pm$ SEM of biological replicates, unpaired two-tailed t-test was performed to assess statistical significance.

j. Pie chart showing the proportions of strongly/mildly up- and down-regulated gene sets related to IL-6 signaling, based on GSEA-derived normalized enrichment scores (NES) determined from the Visium data in **Figure 4a**, comparing AT2 cell-enriched spots in metastasis-vicinity *vs* metastasis-distal regions.

k. Normalized *Fasn* expression levels in AT2-enriched spots within metastasis-vicinity and metastasis-distal regions, based on the Visium data in **Figure 4a**. The violin plots show normalized *Fasn* expression-level distributions (in units of counts per 10,000 reads, CP10k) for each region type, while the solid lines indicate the median *Fasn* expression levels within each of the region types, and dash lines indicate the quartiles. The p-value reported in the figure originates from the results of differential expression analysis comparing AT2 cell-enriched spots in metastasis-vicinity vs metastasis-distal regions, performed within the *Seurat* framework (see Methods), and is subject to FDR adjustment using the Benjamini-Hochberg approach (considering all genes included in the data set).

l. Western blots of MTHFD1L and β-Actin in cell lysates extracted from wild type (WT), knockout control (KO Ctrl), and two independent MTHFD1L knockout 4T1 cell lines (KO1 and KO2).

m. ^{12}C-formate (M+0) and ^{13}C-formate (M+1 isotopologue) secretion rates in 4T1 WT, MTHFD1L KO-Ctrl, MTHFD1L KO_1 and MTHFD1L KO_2 cells cultured with $^{13}C_3$-serine tracer. Secretion rates are shown as mean ± SEM of three biological replicates per isotopologue and cell line. The negative values for the M+0 isotopologue in the case of both MTHFD1L KO_1 and MTHFD1L KO_2 cells indicate net uptake (rather than secretion) of ^{12}C-formate present in the media.

n. Western blots of SREBP-1 and β-Actin in cell lysates extracted from recombinant IL-6 (2ng/mL), and from recombinant IL-6 (2ng/mL) combining with JAK inhibitor Fedratinib (0.5µM) treated *ex vivo* cultured Balb/c mouse AT2 cells for 24 hours. Full length and cleaved mature SREBP-1 are marked in the blots.

Supplementary Figure 5

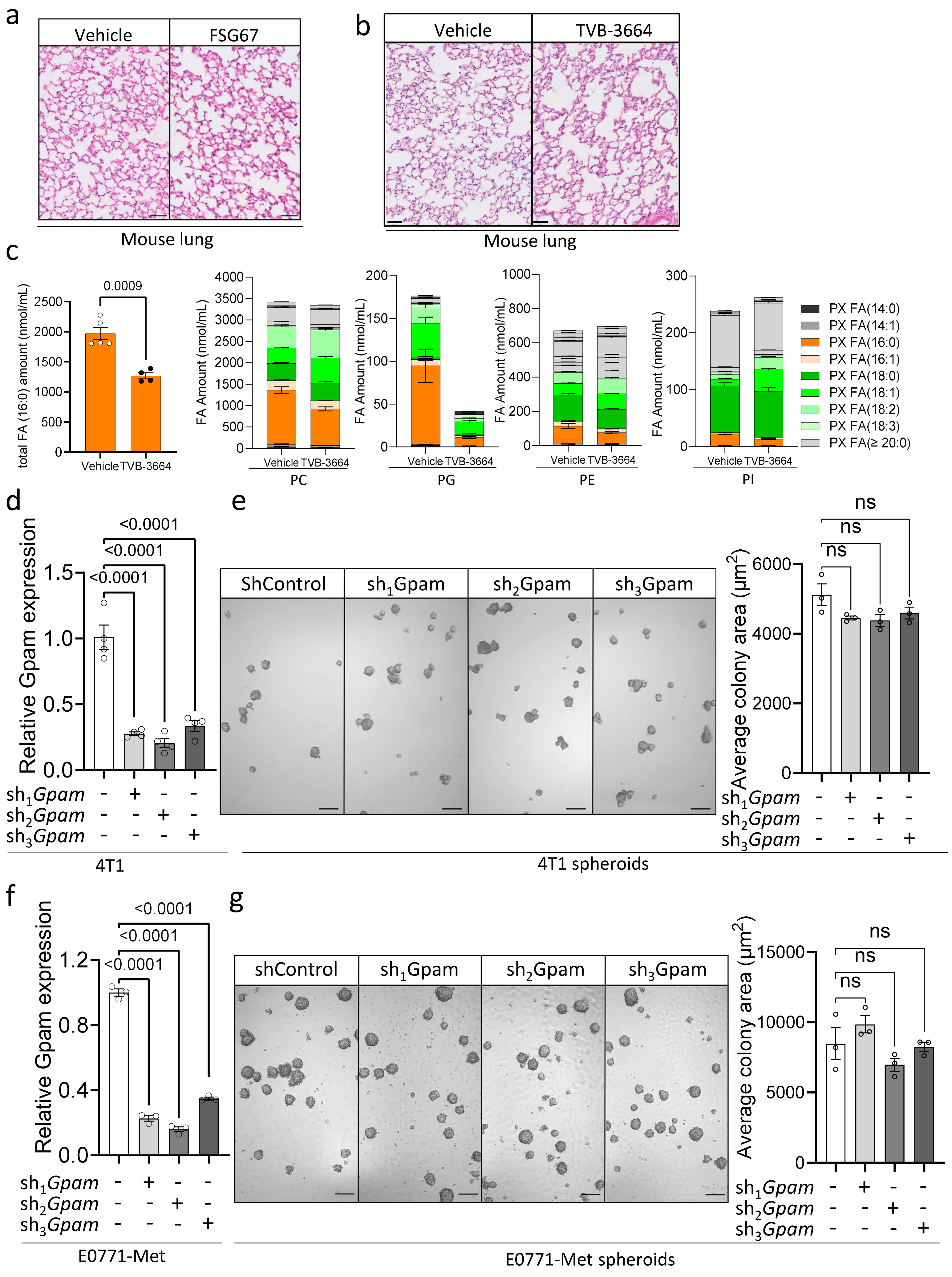

Supplementary Figure 5 *Gpam* knockdown in cancer cells does not affect 3D spheroid growth *in vitro*

a. H&E staining showing the morphology of lungs of Balb/c mice treated with vehicle and FSG67 (5mg/kg body weight daily i.p.) for 19 days.

b. H&E staining showing the morphology of lungs of Balb/c mice treated with vehicle and TVB-3664 (3mg/kg daily oral gavage) for 16 days.

c. Total fatty acid (16:0) content and fatty acid composition of the lipid classes phosphatidylcholine (PC), phosphatidylglycerol (PG), phosphatidylethanolamine (PE) and phosphatidylinositol (PI) in lung interstitial fluid of BALB/c mice treated with vehicle and FASN inhibitor TVB-3664. Data are presented as mean ± SEM (*n* = 5 for vehicle and 4 for TVB-3664 treatment). Statistical significance was assessed using an unpaired two-tailed *t*-test.

d. RT-qPCR was used to measure efficiency of *Gpam* knockdown in 4T1 cells. Knockdown was performed by using three independent shRNAs. Data are presented as mean± SEM of four biological replicates. Ordinary one-way ANOVA with Dunnett's multiple comparisons test was performed to assess statistical significance.

e. 4T1 spheroids grown upon the knockdown of *Gpam* by using three independent shRNAs. Representative pictures are depicted on the left. Scale bars represent 200μm. Average colony areas are presented as mean± SEM of three biological replicates. Ordinary one-way ANOVA with Dunnett's multiple comparisons test was performed to assess statistical significance.

f. RT-qPCR was used to measure efficiency of *Gpam* knockdown in E0771-Met cells. Knockdown was performed by using three independent shRNAs. Data are presented as mean± SEM of three biological replicates. Ordinary one-way ANOVA with Dunnett's multiple comparisons test was performed to assess statistical significance.

g. E0771-Met spheroids grown upon the knockdown of *Gpam* by using three independent shRNAs. Representative pictures are depicted on the left. Scale bars represent 200μm. Average colony areas are presented as mean± SEM of three biological replicates. Ordinary one-way ANOVA with Dunnett's multiple comparisons test was performed to assess statistical significance.

Supplementary Figure 6

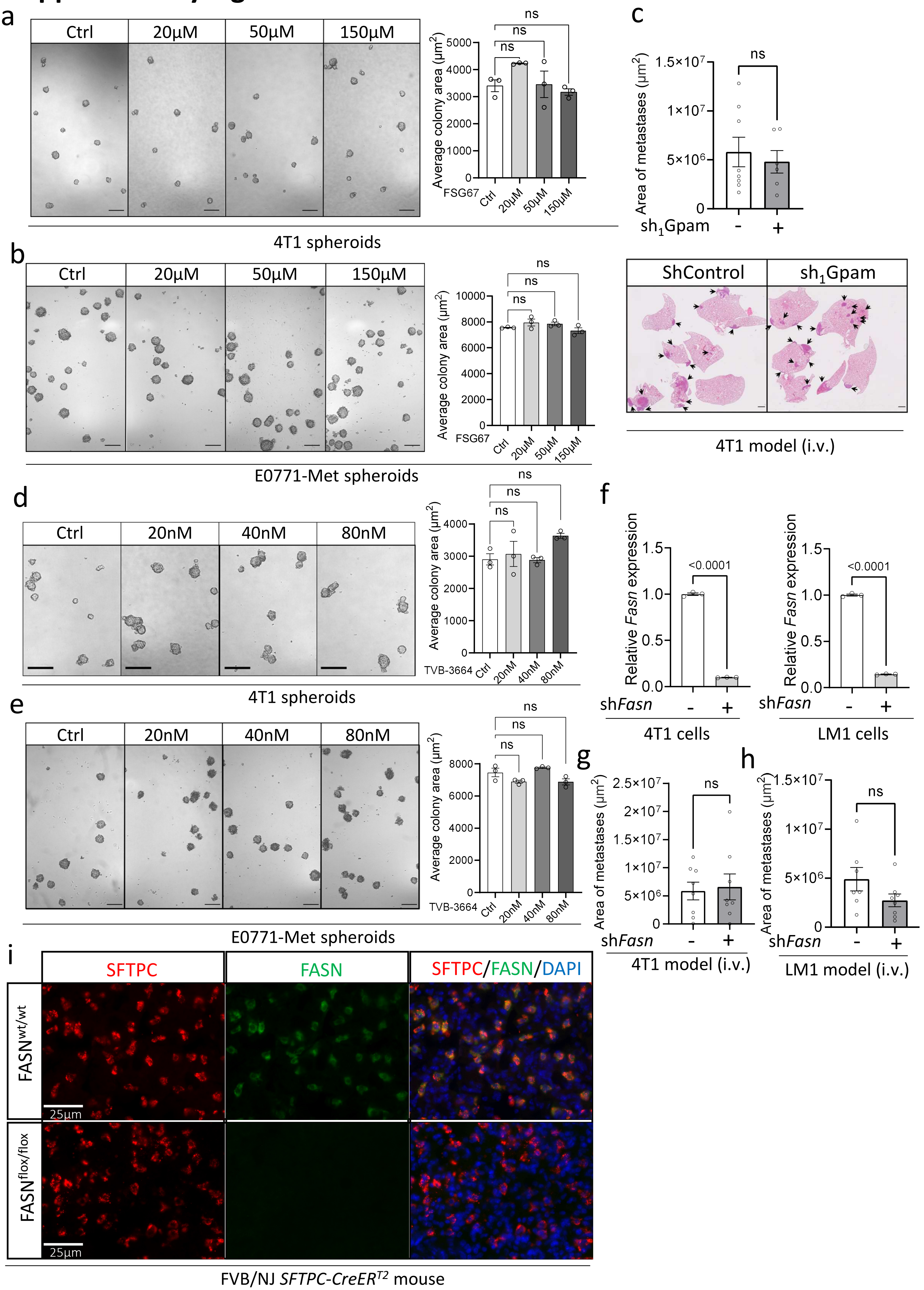

Supplementary Figure 6 GPAM and FASN inhibition in cancer cells does not affect metastasis formation *in vitro* and *in vivo*

a. b. 4T1 (a) and E0771-Met (b) spheroids grown upon the treatment of different concentrations (0, 20, 50 and 150μM) of FSG67 for 5 days (n = 3/conditions). Data are presented as mean ± SEM of biological replicates. Representative pictures are depicted on the left. Scale bars represent 200μm. Ordinary one-way ANOVA with Dunnett's multiple comparisons test was performed to assess statistical significance.

c. Representative H&E staining of lung tissues from Balb/c mice i.v. implanted with 4T1 control (n = 8) and 4T1 *Gpam* knockdown cells (n = 6). Arrows indicate metastatic lesions. Scale bars represent 1mm. The area of metastases is presented as mean + SEM, unpaired two-tailed t-test was performed to assess statistical significance.

d. 4T1 spheroids grown upon the treatment of different concentrations (0, 20, 40 and 80nM) of TVB-3664 for 5 days (n = 3/conditions). Representative pictures are depicted. Scale bars represent 200μm. Data are presented as mean ± SEM of biological replicates. Ordinary one-way ANOVA with Dunnett's multiple comparisons test was performed to assess statistical significance.

e. E0771-Met spheroids grown upon the treatment of different concentrations (0, 20, 40 and 80nM) of TVB-3664 for 5 days (n = 3/conditions). Representative pictures are depicted. Scale bars represent 200μm. Data are presented as mean ± SEM of biological replicates. Ordinary one-way ANOVA with Dunnett's multiple comparisons test was performed to assess statistical significance.

f. RT-qPCR was used to measure efficiency of *Fasn* knockdown in 4T1 (left) and LM1 (right) cells. Knockdown was performed by using shRNAs. Data are presented as mean± SEM of three biological replicates, unpaired two-tailed t-test was performed to assess statistical significance.

g. Quantification of metastasis area of lung tissues from Balb/c mice i.v. implanted with 4T1 control and 4T1 *Fasn* knockdown cells (n = 8). The area of metastases is presented as mean + SEM, unpaired two-tailed t-test was performed to assess statistical significance.

h. Quantification of metastasis area of lung tissues from Balb/c mice i.v. implanted with LM1 control (n = 7) and LM1 *Fasn* knockdown cells (n = 8). The area of metastases is presented as mean + SEM, unpaired two-tailed t-test was performed to assess statistical significance.

i. Immunofluorescence staining for SFTPC (red), FASN (green) and DAPI (blue) in lungs of FASN wild type (*Sftpc-CreER*^{T2};FASN$^{wt/wt}$) and FASN AT2 conditional knockout (*Sftpc-CreER*^{T2}; FASN$^{flox/flox}$) mice. Scale bars represent 25μm.
